# Menin inhibition suppresses castration-resistant prostate cancer and enhances chemosensitivity

**DOI:** 10.1038/s41388-021-02039-2

**Published:** 2021-10-28

**Authors:** Chaïma Cherif, Dang Tan Nguyen, Clément Paris, Thi Khanh Le, Thibaud Sefiane, Nadine Carbuccia, Pascal Finetti, Max Chaffanet, Abdessamad El kaoutari, Julien Vernerey, Ladan Fazli, Martin Gleave, Mohamed Manai, Philippe Barthélémy, Daniel Birnbaum, François Bertucci, David Taïeb, Palma Rocchi

**Affiliations:** 1 Predictive Oncology Laboratory, Centre de Recherche en Cancérologie de Marseille, Inserm UMR 1068, CNRS UMR 7258, Institut Paoli-Calmettes, Aix-Marseille University, 27 Bd. Leï Roure, F-13009 Marseille, France; 2 Laboratory of Biochemistry and Molecular Biology, Science University of Tunis, 2092 El Manar, Tunis, Tunisia; 3 The Vancouver Prostate Centre, University of British Columbia, Vancouver, Canada; 4 ARNA Laboratory, INSERM U1212, CNRS UMR 5320, University of Bordeaux, F-33076 Bordeaux, France; 5 Biophysics and Nuclear Medicine Department, La Timone University Hospital, European Center for Research in Medical Imaging, Aix-Marseille University, F-13005 Marseille, France

**Keywords:** Cancer genetics, Hormone receptors

## Abstract

Disease progression and therapeutic resistance of prostate cancer (PC) are linked to multiple molecular events that promote survival and plasticity. We previously showed that heat shock protein 27 (HSP27) acted as a driver of castration-resistant phenotype (CRPC) and developed an oligonucleotides antisense (ASO) against HSP27 with evidence of anti-cancer activity in men with CRPC. Here, we show that the tumor suppressor Menin (*MEN1*) is highly regulated by HSP27. Menin is overexpressed in high-grade PC and CRPC. High *MEN1* mRNA expression is associated with decreased biochemical relapse-free and overall survival. Silencing Menin with ASO technology inhibits CRPC cell proliferation, tumor growth, and restores chemotherapeutic sensitivity. ChIP-seq analysis revealed differential DNA binding sites of Menin in various prostatic cells, suggesting a switch from tumor suppressor to oncogenic functions in CRPC. These data support the evaluation of ASO against Menin for CRPC.

## Graphical abstract

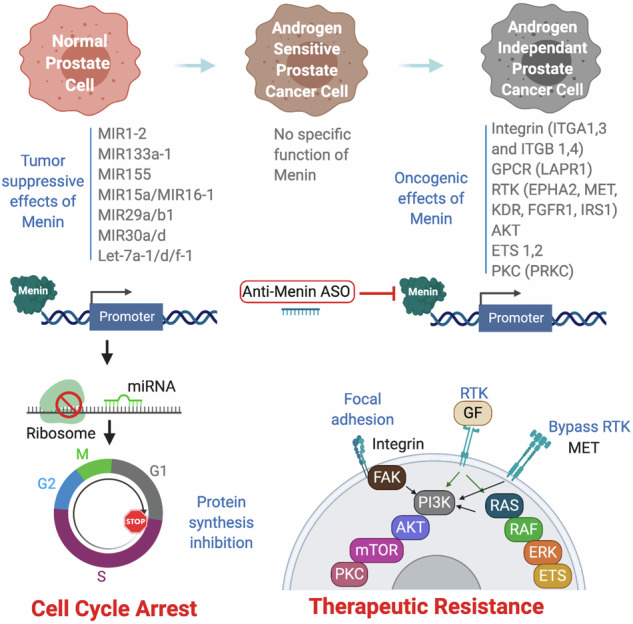

## Introduction

Prostate cancer (PC) is one of the most common cancers in European and American countries. Most of the metastatic patients will initially respond to androgen deprivation therapy (ADT). Still, the disease will progress after several years to castration-resistant PC (CRPC), which remains a challenging therapeutic situation for clinicians [1]. Previously, we identified the stress-activated cytoprotective chaperone heat shock protein 27 (HSP27) to be highly overexpressed in CRPC, supporting tumor cell survival and plasticity *via* various pathways. HSP27 inhibition *via* ASO and small interference RNA (siRNA) has antitumor effects and enhances chemotherapy efficacy [2–4]. Recently, we described a wide diversity of new HSP27 functions that can play critical roles in PC tumorigenesis and therapeutic resistance [5]. We found that HSP27 regulates its protein partner expression at the post-translational level, such as the eukaryotic initiation factor 4E (eIF4E) and translationally controlled tumor protein (TCTP), which were demonstrated to be rapidly ubiquitinated and degraded by the proteasome in HSP27 absence [6, 7].

To establish a more comprehensive network of HSP27-regulated proteins in PC, we employed a large-scale proteomic approach and identified Menin as a novel highly regulated HSP27 client protein in CRPC. Menin is a scaffold protein encoded by the multiple endocrine neoplasia type 1 (*MEN1*) gene. *MEN1* gene mutations cause a hereditary autosomal dominant tumor syndrome (MEN1 syndrome), leading to tumor formation in multiple endocrine organs [8, 9]. The majority of mutations found in MEN1 disease are nonsense and frameshift mutations leading to the inactivation of Menin [10]. In these patients, the coexistence of germline mutations and loss of heterozygosity (LOH) in tumor tissue for tumorigenesis demonstrates that the *MEN1* gene acts as a classic tumor suppressor [11]. Menin was involved in the regulation of multiple important signaling pathways at a transcriptional level via direct and indirect interactions with epigenetic factors such as KMT2A/2B, EZH2, and transcription factors including JunD, Smad1/3/5, and P53 [9, 12–17]. Menin also regulates cell proliferation, apoptosis, and maintenance of genome integrity. Although Menin acts as a tumor suppressor in endocrine tissues [18, 19], recent studies have shown that Menin could promote tumorigenesis in various neoplasms [9, 20]. Recently, in a small series of cases, *MEN1* was found overexpressed in CRPC [21]. The mixed-lineage leukemia (MLL) complex acts as a co-activator of androgen receptor (AR) signaling, which plays a critical role in PC development and notably for CRPC progression [22]. AR directly interacts with the MLL complex *via* the Menin-MLL subunit [21]. Recently, small-molecule inhibitors of the Menin-MLL interaction were developed [23, 24]. Blockade of the Menin-MLL interaction inhibited AR signaling and CRPC growth in AR-dependent models [21]. Although these inhibitors represent a useful compound for reversing the oncogenic activity of MLL, they were not suitable for in vivo efficacy studies because of their modest cellular activity and very poor metabolic stability [23, 25, 26]. In CRPC, the Menin inhibitors developed inhibit Menin-MLL only in AR-expressing cells with no effect in AR-depleted cells such as PC-3 and DU-145.

This work aims to define the broad functions of Menin in PC. We identify Menin as the most highly HSP27-regulated protein in CRPC. The analysis of protein and mRNA expression of Menin in an extensive collection of human samples revealed that high expression was associated with tumor aggressiveness features, such as metastatic stage, hormone resistance, higher tumor grade, and worse survival. We developed a specific Menin inhibitor, restoring therapeutic sensitivity in CRPC models (Menin-ASO (WO2017134252 (A1), 2017) [27]. Chromatin immunoprecipitation combined with next-generation sequencing (ChIP-seq) demonstrated how Menin acquires oncogene functions during PC progression and confers resistance to therapies. Our study reveals that Menin is involved in resistance to PC treatment by activating PI3K/AKT pathway and Menin inhibition restores chemotherapeutic sensitivity. It reveals that Menin drives resistance to PC treatments through activation of the PI3K/AKT pathway and our Menin inhibitor restores chemotherapeutic sensitivity. Our results provide a rationale for targeting Menin to improve the therapeutic outcomes for CRPC patients.

## Results

### Hsp27 interacts and protects Menin from degradation by the ubiquitinal-proteasome system

**Fig. 1:**
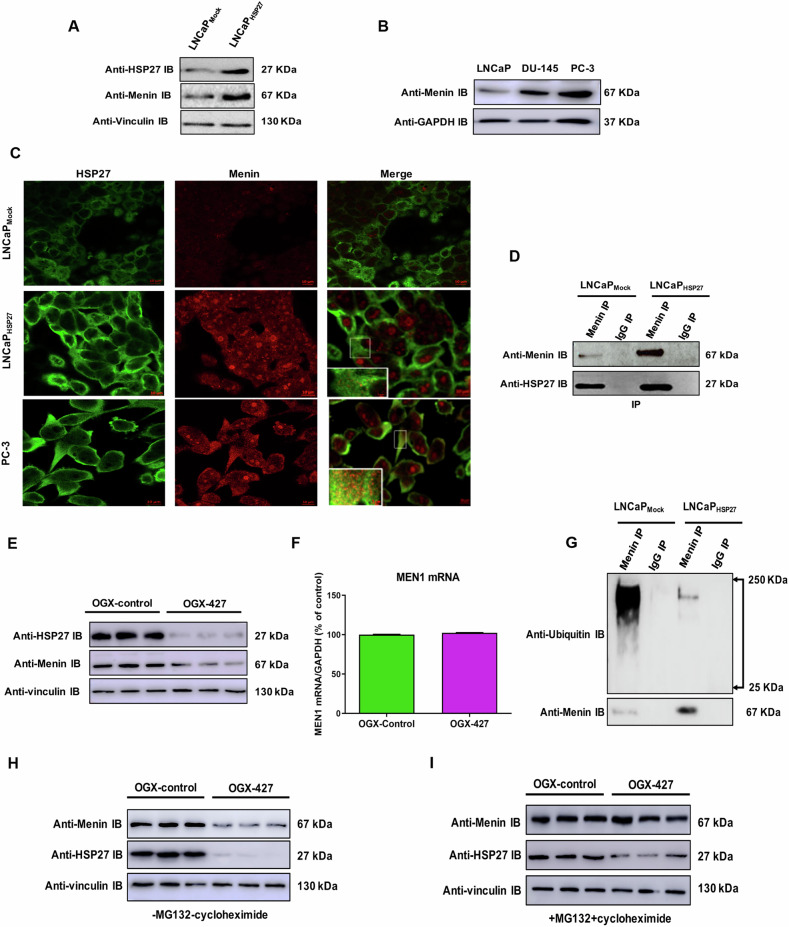
Menin as a novel HSP27 ubiquitin-proteasome regulated partner in CRPC. **A** HSP27, Menin, and vinculin protein levels analyzed were by western blot in CSPC (LNCaP-Mock) and CRPC (LNCaP-HSP27) models. **B** Menin and GAPDH western blot in CSPC (LNCaP) and AIPC (DU-145, PC-3) cell lines. **C** LNCaP-Mock, LNCaP-HSP27, and PC-3 cells were fixed and immunostained using Menin (red immunofluorescence) and HSP27 (green immunofluorescence) antibodies. Yellow puncta represented colocalization. **D** LNCaP-Mock and LNCaP-HSP27 cell lysates were used to immunoprecipitate (IP) Menin using mouse anti-Menin or mouse anti-immunoglobulin (IgG) antibodies. Total cell lysate (TCL) represents proteins from LNCaP-Mock *versus* LNCaP-HSP27 cells were extracted and blotted with anti-Menin or anti-HSP27 antibodies. **E** PC-3 cells were treated with OGX-427 or OGX-control for two days and then harvested for protein extraction and tested by western blot analysis using an anti-Menin, anti-HSP27, and anti-vinculin antibodies. **F** Menin and GAPDH levels were analyzed by quantitative reverse transcription-PCR (qRT-PCR) on total RNAs extracted from a culture of PC-3 treated with OGX-427 or OGX-control. Menin mRNA levels were analyzed after normalization to GAPDH RNA levels. Results are expressed as the percentage of PC-3 cells transfected with the OGX-control (100%). **G** Protein lysates from LNCaP-Mock and LNCaP-HSP27 cells were used to immunoprecipitate (IP) Menin, followed by western blotting with an anti-ubiquitin (Ub) antibody. **H**, **I** PC-3 cells were treated with OGX-427 or OGX-control for 2 days. After transfection, cells were harvested for protein extraction or pretreated with cycloheximide (10 μg/ml) followed by MG-132 (10 μmol/l) for 48 h and tested by western blot analysis using an anti-Menin, anti-HSP27, and anti-vinculin antibodies. HSP27, heat shock protein 27.

Previously, we used LNCaP stably transfected with lentivirus expressing HSP27 (LNCaP-HSP27) to show that overexpression of this chaperone inhibits androgen withdrawal-induced apoptosis and promotes CRPC [3, 6, 7]. To obtain a large-scale overview of HSP27-regulated proteins driving CRPC, we performed a proteomic analysis in CRPC LNCaP-HSP27 [3] and castration-sensitive prostate cancer (CSPC) LNCaP-Mock models. Accordingly, we identified a total of 1157 proteins in LNCaP-HSP27 and 1418 proteins in LNCaP-Mock with an overlap of 818 proteins. To identify HSP27-regulated proteins driving CRPC progression, the list of identified proteins (Table S1) was analyzed using enrichment of biological pathways supplied by the KEGG database. This analysis showed the involvement of HSP27 in several pathways such as apoptosis, VEGF, RAS, MAPK, metabolism, cell cycle, TP53, prostate cancer, PI3K/AKT, and transcriptional misregulation in the cancer pathway (Table S2). Because it is the most highly regulated pathway, we focused our attention on the “transcriptional misregulation in cancer pathway” (hsa05202), containing three identified proteins (DEAD-Box Helicase 5, DDX5; eyes absent 1 protein, EYA1, and Menin) present in CRPC model (LNCaP-HSP27) and not detected in CSPC model (LNCaP-Mock) (not shown). We decided to study Menin further because it was the most highly Hsp27 regulated protein (eight Menin isoforms and O00255 was the most regulated with no expression in the CSPC model (LNCaP-Mock) (Table S3). Furthermore, western blot (WB) analysis in PC models showed that Menin expression was 4-fold higher in CRPC (LNCaP-HSP27) compared to CSPC (LNCaP-Mock) model (Figs. 1A and S1A) and 2-fold higher in AI PC-3 and DU-145 cell lines than in CSPC LNCaP cell lines (Figs. 1B and S1B). To better understand HSP27 role in Menin regulation, we examined whether HSP27 colocalized and interacted with Menin using immunofluorescence (IF) and co-immunoprecipitation (co-IP). Confocal microscopy showed that Menin (red) colocalized with HSP27 (green) in the cytoplasm of LNCaP-Mock, LNCaP-HSP27, and PC-3 cells (Fig. 1C). Co-IP experiment using cell lysates from LNCaP-Mock and LNCaP-HSP27 cells showed an interaction between HSP27 and Menin (Figs. 1D and S1C). To define HSP27 role on the regulation of Menin expression, we used the modified ASO OGX-427 (Apatorsen) that was already validated and tested in clinical trials [28] to the knock-down expression of HSP27 in PC-3 cells. Figures 1E and S1D show that OGX-427 caused a 56% decrease in Menin protein level in PC-3 cells, with no effect on the mRNA level (Fig. 1F), suggesting a post-translational regulation. Then, we evaluated if this effect is involved in the chaperone activity of HSP27. As shown in Fig. S1E, Menin level decreased in a time-dependent manner when cells were treated with cycloheximide (CHX). Meanwhile, it was stabilized when cells were treated with both CHX and a proteasome inhibitor (MG132), indicating that the proteasome pathway regulates Menin’s stability. We tested the HSP27 effect on Menin ubiquitination rates and subsequent proteasomal degradation using LNCaP-Mock and LNCaP-HSP27 cells. As shown in Fig. 1G, HSP27 overexpression decreased the ubiquitination level of Menin as indicated by the ladder of high-molecular-weight species, which is a characteristic of polyubiquitinated proteins. To determine whether the ubiquitination of Menin results in its proteasomal degradation, Menin’s half-life was measured after 48 h of OGX-427 treatment in the presence or absence of the proteasome inhibitor MG132 and protein synthesis inhibitor cycloheximide. WB analysis showed that proteasome inhibitor MG132 (+cycloheximide) reversed the effect of OGX-427 and stabilized Menin, indicating that a decrease in Menin protein level after OGX-427 treatment is mediated by proteasomal degradation (Fig. 1H and I).

### Menin expression correlates with prostate cancer progression and hormone resistance

**Fig. 2:**
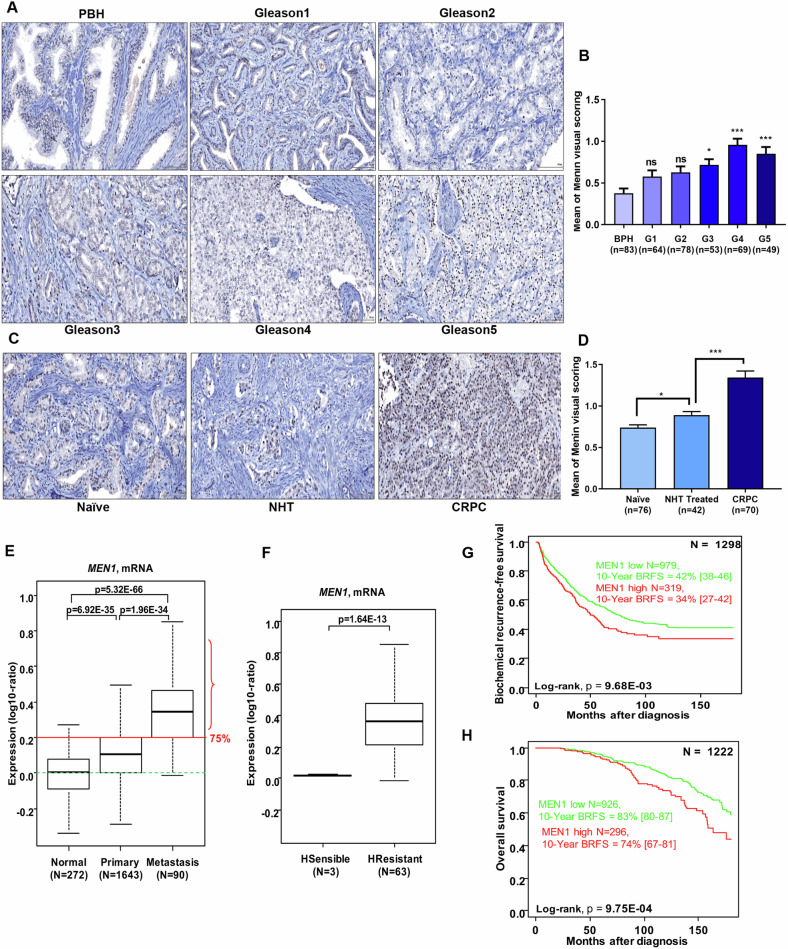
Menin expression correlates with prostate cancer progression, hormone resistance, and associated with poor prognosis. **A** Representative microscopic fields of Menin immunostaining in Gleason tissue microarray (TMA). **B** Means of Menin expression staining in Gleason TMA composed from 198 patients (2 cores from each patient): 41 BHP patients (SEM: 0.056) and 157 PC patients in different Gleason G1 (SEM: 0.073), G2 (SEM: 0.071), G3 (SEM: 0.068), G4 (SEM: 0.075), and G5 (SEM 0.080). **C** Representative microscopic fields of Menin immunostaining in Neoadjuvant Hormonal Therapy (NHT) TMA. **D** Means of Menin expression in NHT TMA composed from 94 patients (2 cores from each patient): 38 no treated patients (naïve) (SEM: 0.034), 21 patients treated with NHT (SEM: 0.043), and 35 patients CRPC (SEM: 0.078). Specimens were graded from 0 to +3 intensity, representing the range from no staining to heavy staining by visual scoring and automated quantitative image analysis by Image-pro Plus software. **E** Box-plot of *MEN1* mRNA expression levels in 272 “normal” samples (normal tissue and prostate benign hypertrophia), 1643 PC primary tumor samples, and 90 PC metastatic samples collected from public data sets. The *p*-values are for the Student *t*-test. **F** Similar to (**E**), but for 3 hormone-sensitive *versus* 63 hormone-resistant metastatic samples. **G** Kaplan–Meier biochemical relapse-free survival (BRFS) and **H** overall survival (OS) curves in the “*MEN1*-low” class (green curve) *versus* “*MEN1*-high” class (red curve). The *p*-value is for the log-rank test.

To determine the clinical value of Menin expression in PC, we analyzed Menin immunoexpression on human samples obtained at a different stage of disease progression. Menin IHC was performed on two different tissue microarrays (TMAs) constructed at the Vancouver Prostate Center. Analysis of the first TMA composed of 198 patients showed that Menin expression correlated positively with Gleason grade progression. The mean intensity of Menin visual scoring in different ISUP2014 Gleason grades (Benign, G1, G2, G3, G4, and G5) was 0.37, 0.58, 0.63, 0.71, 0.95, and 0.85, respectively (Fig. 2A and B). Furthermore, Menin was highly overexpressed in high Gleason grades (3, 4, and 5) compared with benign samples: *p* = 2E−04 for benign *vs* G3 and *p* < 1E−04 for benign *vs* G4 and G5 samples. The second TMA was composed of samples from 94 patients treated with neoadjuvant hormonal therapy (NHT) and revealed that Menin expression increased in patients that received NHT treatment to become overexpressed in 90% of CRPC patients. The mean intensity of Menin in hormone-naive, NHT-treated, and CRPC were 0.74, 0.89, and 1.34, respectively (Fig. 2C and D). The two TMA was constructed by using two cores from each patient.

We then extended our expression analysis to *MEN1* mRNA expression in a pooled series of 2081 clinical tissue samples publicly available, including 272 “normal” samples (normal tissue and benign prostate hyperplasia: BPH), 1643 PC primary tumor samples, and 90 PC metastatic samples (Supplementary methods and Table S4). Accordingly, the upregulation of *MEN1* mRNA expression was strongly associated with PC metastasis and hormone therapy resistance (Fig. 2E, F and Table S5). Moreover, evaluation of the prognostic value of *MEN1* expression in the 1643 primary tumor samples has shown that *MEN1* expression is independently associated with worse biochemical relapse-free survival (BRFS) and overall survival (OS) (Fig. 2G, H and Table S6).

Altogether, these data indicated that *MEN1* is overexpressed in PC samples. High expression is associated with aggressiveness factors such as metastatic stage, hormone resistance, higher tumor grade and is independently associated with worse BRFS and OS.

### Menin silencing inhibits Hsp27-mediated cytoprotective effects and restores treatment sensitivity

**Fig. 3:**
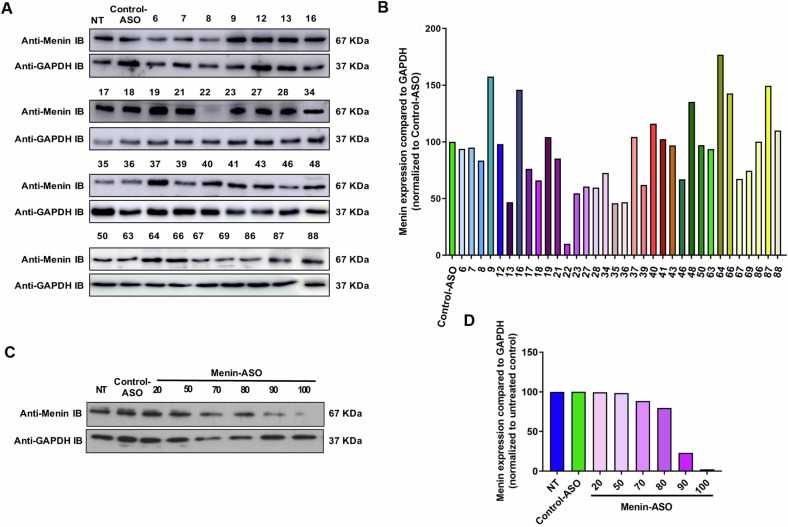
Menin-ASO causes 90% abrogation of Menin expression. **A** PC-3 cells were treated with 100 nM of differents ASOs or control-ASO. Two days after the second transfection, proteins were extracted and analyzed by western blot. **B** Bands were quantified using ImageJ software normalized to GAPDH protein levels. Data were normalized to control-ASO (100%). **C** PC-3 cells were treated with indicated concentrations of ASOs or control-ASO. Two days after the second transfection, proteins were extracted and analyzed by western blot. **D** Bands were quantified using ImageJ software normalized to GAPDH protein levels. Data were normalized to untreated-control (100%).

**Fig. 4:**
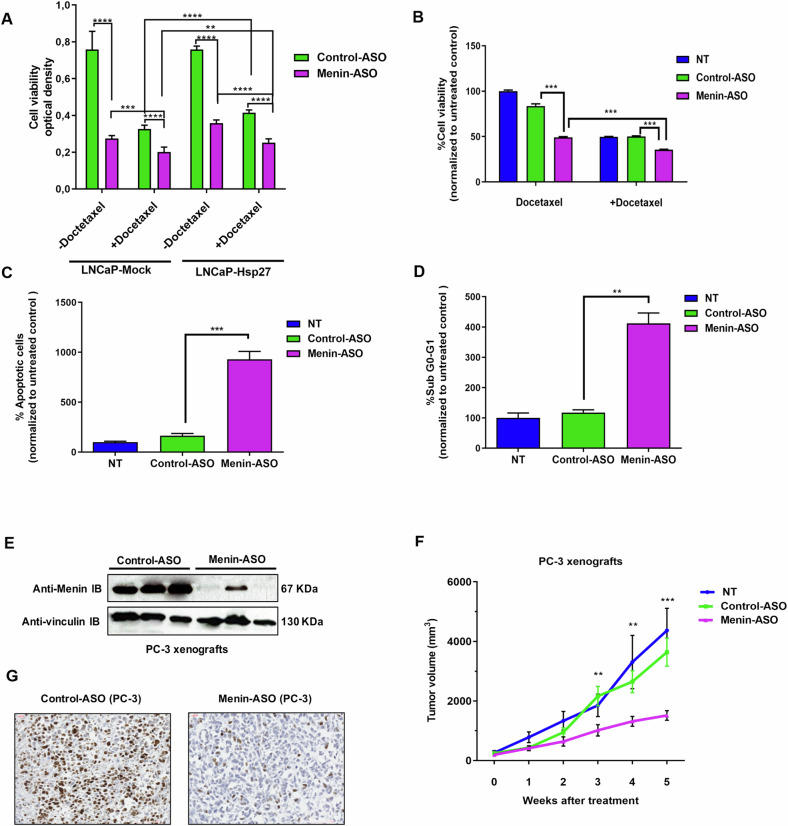
Menin is involved in cytoprotection induced by HSP27 and Menin-ASO inhibits PC cell growth and restores treatment sensitivity. **A** LNCaP-Mock and LNCaP-HSP27 cells were treated with 100 nM Menin-ASO or control-ASO. After 2 days, docetaxel was added for 24 h. Cell viability was determined using the MTT test. Data are shown as mean ± SEM, *n* = 3. Two-tailed, unpaired Student’s *t*-test (****P ≤ 0.0001; ***P ≤ 0.001; **P ≤ 0.01). **B** PC-3 cells were transfected with 100 nM Menin-ASO or control-ASO for two days and treated with docetaxel for 24 h. Cell viability was determined using the MTT test. Data were normalized to untreated cells, and are shown as mean ± SEM, *n* = 3. Two-tailed, unpaired Student’s *t*-test (***P ≤ 0.001; **P ≤ 0.01). **C** PC-3 cells were transfected with 100 nM Menin-ASO or control-ASO for 2 days after the second transfection. Apoptosis was assessed by Annexin V binding. Flow cytometry was used to quantify the apoptotic rates (**D**) and to quantify the percentage of PC-3 cells in each cell cycle phase. Data were normalized to untreated cells and are shown as mean ± SEM, *n* = 3. Two-tailed, unpaired Student’s *t*-test (***P ≤ 0.001). **E** Western blot analysis of Menin expression in PC-3 xenografts after 7 days of treatment with Menin- or control-ASO. **F** Mice bearing AIPC (PC-3) tumors were randomly selected for treatment with Menin- or control-ASO. When PC-3 tumors reached 50 mm^3^, 12.5 mg/kg/mouse of Menin- or control-ASO were injected intraperitoneally (i.p.) daily for 5 weeks in animals receiving ASO monotherapy. Tumor volume was measured once weekly and calculated by the formula length × width × depth × 0.5236. Data show mean ± SEM. Two-tailed, unpaired Student’s *t*-test, *P ≤ 0.05, **P ≤ 0.01, ***P ≤ 0.001. **G** Ki-67 IHC staining of tumor tissues to access tumor cell proliferation.

To determine the impact of Menin silencing in CRPC cells, we first designed 91 ASOs targeting the entire Menin mRNA. Among these, 34 were selected for testing in PC-3 cells because of their low GC percentage and 100% specificity to Menin (Table S7). Menin-ASO22 was the most effective ASO causing a 90% abrogation of Menin expression at 100 nM (Fig. 3A, B). Dose-dependent ASO treatment allowed us to determine the minimum efficient dose (MED) of Menin-ASO at 100 nM (Fig. 3C, D). Menin knockdown using ASO22 (Menin-ASO) in CRPC LNCaP-HSP27 or CSPC LNCaP-Mock reversed the cytoprotection to docetaxel treatment normally conferred by HSP27 overexpression (Fig. 4A*;* ****P ≤ 0.001; lanes 3–7, **P ≤ 0.001; lanes 4–8). This result revealed that Menin is an important effector of HSP27 cytoprotective function. Menin-ASO treatment also caused a significant reduction in AI PC-3 cell growth (51%) compared to control cells treated with scrambled ASO (***P ≤ 0.001; lanes 2–3) (Fig. 4B) with a synergistic effect with docetaxel sensitivity by up to 13% (***P ≤ 0.001; lanes 3–6) (Fig. 4B). Flow cytometry analysis of annexin V expression demonstrated that Menin-ASO increased apoptosis (5–6-fold) of AI PC-3 cells (***P ≤ 0.001) compared to control-ASO (Fig. 4C). Interestingly, this effect was found only in AIPC cells since Menin-ASO did not induce apoptosis in normal prostate model PNT1A (Figure S2A), highlighting the potential switch in Menin functions during PC progression. Furthermore, cell cycle analysis showed that the fraction of AI PC-3 cells undergoing apoptosis (sub-G0/G1 fraction) was higher (3–4-fold) with Menin-ASO compared to control-ASO (**P ≤ 0.01) (Fig. 4D).

In conclusion, Menin knockdown using ASO22 (Menin-ASO) partially mimicked the effects of HSP27 knockdown that were previously described, suggesting that Menin is an important mediator of the effect of HSP27 in CRPC.

We next evaluated the impact of its silencing using ASO treatment in the AI PC-3 xenograft model. Five mice from each group were sacrificed after one week of ASO treatment. Tumors were collected, and Menin protein level was analyzed by WB. As shown in Fig. 4E, a drastic decrease in Menin expression was observed in the Menin-ASO group (86%) compared to the control-ASO group (Figure S2B). Treatment with Menin-ASO decreased tumor volume from weeks 3 to 5 (***P ≤ 0.001) (Fig. 4F). At sacrifice time, tumor volume was >2-fold higher in the control-ASO treated group (3640.5 mm^3^ +/− 470,538 mm^3^) compared to the Menin-ASO treated group (1514 mm^3^ +/− 163,443 mm^3^; ***P ≤ 0.001). Under the experimental conditions described above, no adverse effect was observed (Table 1) Immunohistochemistry (IHC) analysis demonstrated that the proliferation index level (Ki67) in the Menin-ASO treated group was lower than in the control group, suggesting an anti-proliferative effect of Menin-ASO in AI PC-3 xenografted tumors (Fig. 4G).

**Table 1:** Analysis of mice behavior and signs of systemic toxicity during treatment.

	No ASO treatment	Control-ASO	Menin-ASO
Bodyweight	Normal	Normal	Normal
Diarrhea	Neg	Neg	Neg
Respiration	Normal	Normal	Normal
Aggressiveness	Neg	Neg	Neg
Mobility	+/−	+/−	+

Symbols: +, normal; +/−, slightly reduced without affecting the ability to obtain food or water.

### Menin acquires oncogenic functions during PC progression leading to treatment resistance through PI3K/AKT activation

To better understand the oncogenic functions of Menin during PC progression, we profiled Menin chromatin occupancy by ChIP-seq in normal prostate PNT1A (N) and LNCaP (ASPC) and PC-3 (AIPC) PC models. Menin peak location analysis demonstrated that regardless of the disease stage, Menin was predominantly present at transcription start sites (TSS) (−1 kb to +5 kb) (Figure S3A).

**Fig. 5:**
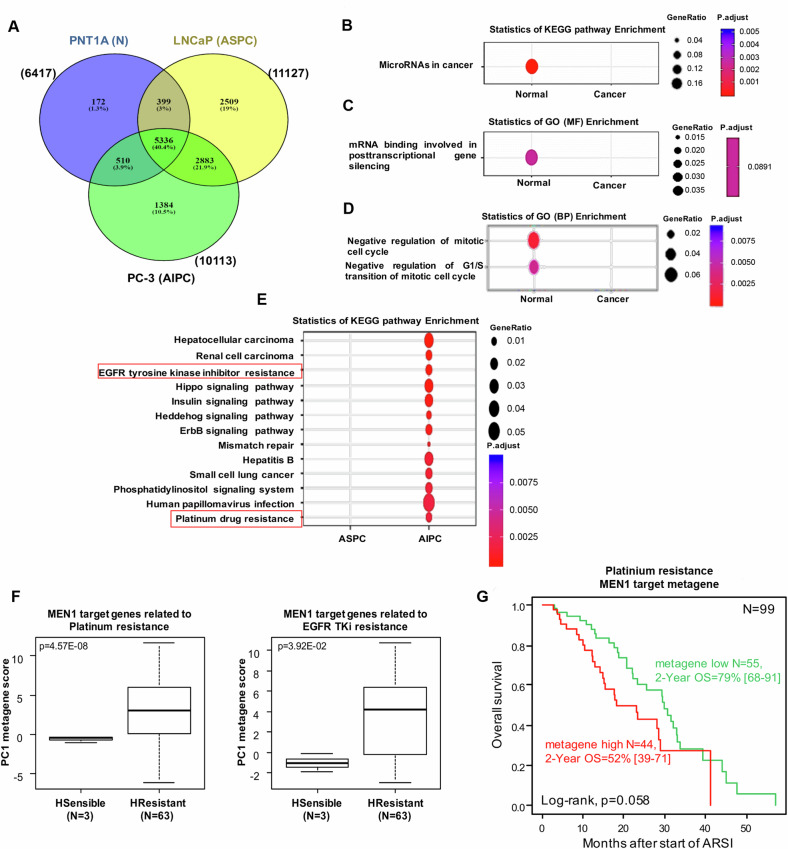
Functional profiles comparison between normal prostate PNT1A (N), LNCaP (ASPC), and PC-3 (AIPC) models. **A** Venn diagram showing the differential of Menin gene binding in PNT1A (N), LNCaP (ASPC), and PC-3 (AIPC) cells. Enrichment of differential bound genes (DBGs) of Menin in PNT1A (N) (172) *versus* PC LNCaP (2509) and PC-3 (1384) models **B** by KEGG pathways **C** by GO terms enrichment of DBGs related to the molecular functions (MF). **D** GO terms enrichment of DBGs using the exclusive list Menin targets genes in LNCaP (ASPC) (2509) *versus* PC-3 (AIPC) (1384) models related to the biological process (BP). **E** KEGG pathways enrichment of DBGs using the full list Menin targets genes in LNCaP (ASPC) (11127) *versus* PC-3 (AIPC) (10113). GeneRatio = amount of DBGs enriched in the pathway/amount of all genes in the annotation gene set, p.adjust ≤ 0.01. GO, gene ontology; KEGG, Kyoto Encyclopedia of Genes and Genomes. **F** Box-plot of the PC1 metagene score based upon expression of Menin target genes of each resistance pathway in 3 hormone-sensitive *versus* 63 hormone-resistant metastatic samples. The p-values are for the Student *t*-test. **G** Kaplan–Meier overall survival curves in 99 patients with mCRPC treated with a first-line next-generation ARSI (abiraterone or enzalutamide) from the Sawyers group’s dataset [29] according to high (red curve) *versus* low (black curve) PC1 metagene score based upon Menin target genes of the Platinum resistance pathway (natural cut-off = 0). The *p*-value is for the log-rank test.

Genomic Regions Enrichment of Annotations Tool (GREAT) determination of the corresponding genomic regions gene demonstrated twice more Menin target genes at TSS genes in cancer LNCaP (11127) and PC-3 (10113) compared to normal PNT1A (6417) cells, suggesting distinct roles of Menin during PC progression (Fig. 5A). As shown in Fig. 5A, Menin bound exclusively to 172 genes in PNT1A (N), 2509 genes in LNCaP (ASPC) and 1384 genes in PC-3 (AIPC). Menin TSS targets are listed in Table S8.

To gain insight into molecular and biological Menin functions in PC progression, ChIP-seq data of each cell line was analyzed concerning ontologies using Kyoto Encyclopedia of Genes and Genomes (KEGG) and Gene Ontology terms (GO), including biological process (BP) and molecular function (MF).

To understand Menin tumor suppressor function in normal cells, we compared the exclusive list of Menin target genes in PNT1A (172) *versus* LNCaP (2509) and PC-3 (1384) (Fig. 5A). KEGG enrichment showed that in PNT1A cells, Menin significantly (*p* = 4.8E–08) regulates 12 microRNAs (MIR1-2; MIR133a-1; MIR155; MIR15a; MIR16-1; MIR29a; MIR29b-1; MIR30a; MIR30d; let-7a-1; let-7d; let-7f-1), widely shown to function as tumor suppressors in PC [21–24] (Fig. 5B, Table S9). This result was supported by GO-MF analysis showing that Menin induces post-transcriptional gene silencing (PTGS) through regulation of microRNAs (*p* = 3E–04) (Fig. 5C). Furthermore, GO-BP enrichment showed that Menin negatively regulates (*p* = 3.92E–06) G1/S mitotic cell cycle transition (Fig. 5D). These findings suggest that these tumor suppressor miRNAs regulation by Menin can induce cell cycle blockade and explain tumor suppressor function of Menin in PNT1A (N) cells.

To understand Menin oncogenic function in CRPC, we compared the exclusive list of Menin target genes in LNCaP (2509) *versus* PC-3 (1384) (Table S8). GO-BP enrichment analysis showed that Menin in AIPC cells acquires significant oncogenic functions (e.g., proliferation, migration) (*p* = 1.5E–05). We then compared the full list of Menin targets genes in LNCaP (11127) *versus* PC-3 (10113) (Fig. 5E, Table S8). KEGG pathway enrichment analysis showed, within the Menin target genes of the AIPC resistant cell line, a significant enrichment in pathways involved in EGFR tyrosine kinase inhibitor resistance (*p* = 6.69E–06) and Platinum drug resistance (*p* = 2.5E–0.4) (Fig. 5E). This enrichment was not observed within the Menin target genes of the ASPC sensitive cell line. Interestingly, the PC1 metagene scores (first component of Principal Component Analysis) applied to Menin target genes of each resistance pathway were higher in castration-resistant *versus* castration-naïve clinical samples of our 66 informative publicly available metastatic samples (*p* = 4.57E–08 for Platinum resistance pathway and *p* = 3.92E–02 for EGFR TKI resistance pathway, Student *t*-test; Fig. 5F), thus validating these results in clinical samples. Such analysis did not apply to the Sawyers’s group dataset [29], including clinical samples from 429 patients with metastatic castration-resistant prostate cancer (mCRPC). However, among the 99 patients treated with a first-line next-generation ARSI (abiraterone or enzalutamide) and with informed overall survival, a higher PC1 metagene score based upon Menin target genes involved in the Platinum resistance pathway was associated with the shorter OS when assessed as continuous value (Cox model: hazards ratio = 3.90 [95%CI 1.15–13.2], *p* = 2.91E–02, Wald test) and as discrete value (*p* = 5.8E–02, log-rank test; Fig. 5G). No significant correlation was found with the metagene based upon Menin target genes involved in the EGFR TKI resistance pathway (data not shown).

**Fig. 6:**
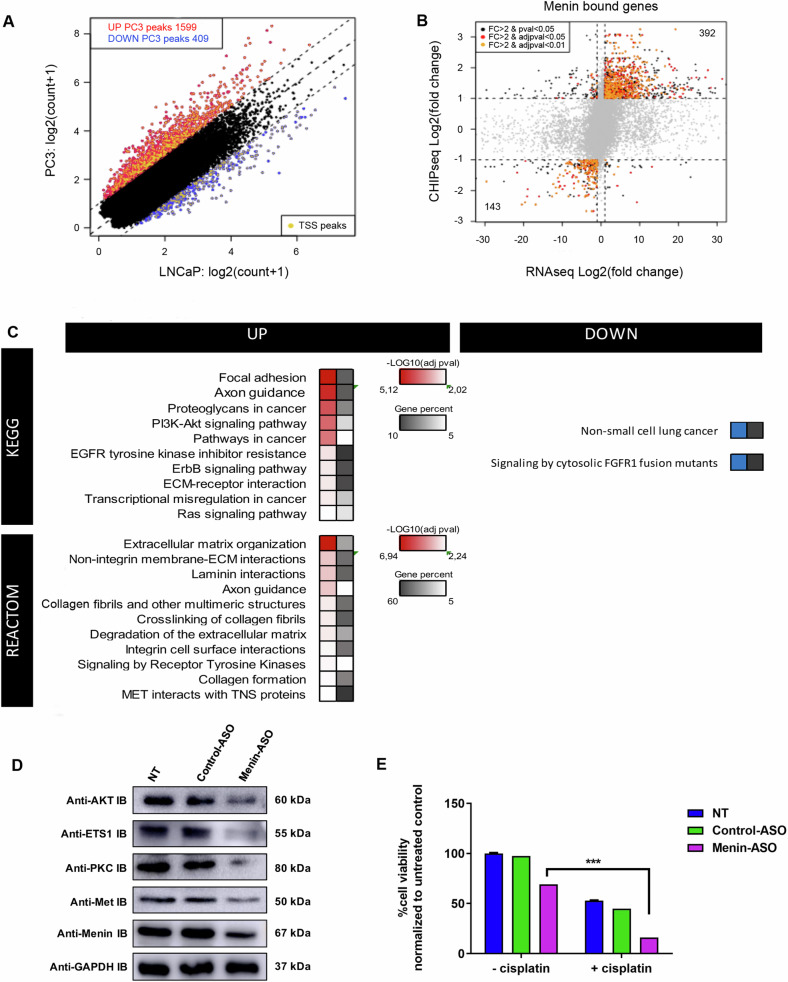
ChIP-seq and RNA-seq comparative analyses and biological validation. **A** Scatter plots showing differential *MEN1* ChIP-seq targets between ASPC (LNCaP) and AIPC (PC-3) models. Peaks with a fold change (FC) > 2 were considered as upregulated peaks (red). Peaks with FC < 0.5 were considered as downregulated peaks (blue). **B** Cross plot between ChIP-seq and RNA-seq comparative analyses. Yellow dots represent genes significantly modulated *p* ≤ 0.05, FC > 2. **C** KEGG and Reactome pathways analysis of Menin-peaks nearby genes. Grayscale values illustrate gene enrichment (i.e., number of genes observed/expected) for each KEGG or Reactome pathways. The red scale indicates the *P*-value (–log10) and greyscale represents gene percent (i.e., % of genes observed/total number of genes within each KEGG or Reactome pathways). **D** Validation of pathways activated by Menin in the AIPC model. PC-3 cells were treated with 100 nM of Menin- or control-ASO and 2 days after second transfection proteins were extracted and analyzed by western blot. **E** PC-3 cells were transfected with 100 nM Menin-ASO or control-ASO for 2 days and treated at 27.9 μM of Cisplatin for 48 h after the second transfection. Cell viability was determined using the MTT test. Data were normalized to untreated cells, and are shown as mean ± SEM, *n* = 3. Two-tailed, unpaired Student’s *t*-test (***P ≤ 0.001).

Our data suggested that Menin acquires CRPC oncogenic function by selectively gene specific-binding. To determine how Menin-specific-binding regulates CRPC progression and treatment resistance, we cross analyzed ChIP-seq data with publically available RNA-seq data from LNCaP and PC-3 (GEO accession GSE59009) [30] (Fig. S3B). ChIP-seq combined with RNA-seq data reveals that Menin upregulated more genes in AIPC (PC-3) model (392) compared to ASPC (LNCaP) model (143). Differentially expressed and Menin-bound genes (DEBGs) were analyzed using clusterProfiler (Fig. 6A, B), showing upregulation of the PI3K/AKT pathway (Fig. 6C, Table S10).

To validate the bioinformatics analysis data, we selected the most important target genes that were identified to be upregulated by ChIP-seq combined with RNA-seq data (Table S10). The western blotting analysis confirmed that AKT3, PRKCA, ETS1, and MET protein levels were downregulated by Menin silencing using ASO (Fig. 6D), supporting that the Menin-driven PIK3-AKT upregulation drives treatment resistance through activation of alternative pathways gene like MET. We used the AIPC (PC-3) model to validate this hypothesis, previously shown to be resistant to Cisplatin [31]. As shown in Fig. 6E, Menin-ASO enhanced cisplatin sensitivity (69%) compared to control-ASO (16.9%) in PC-3 cells (***P ≤ 0.001; lanes 3–6) at the cisplatin concentration of 27.9 μM (Figure S3C).

All these experiments confirm that Menin acts as a tumor suppressor in normal prostate cells due to its miRNA regulation. Menin switches to oncogenic function in the CRPC AR-negative model through its PI3K/AKT pathway activation, leading to resistance to drugs such as EGFR tyrosine kinase inhibitor and Cisplatin.

**Fig. 7:**
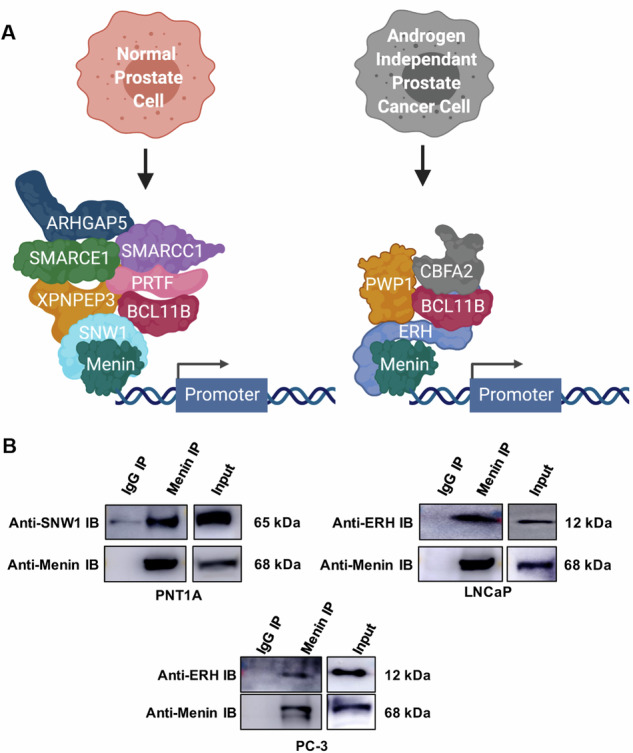
Menin co-regulators. **A** Identification of Menin interacting transcription factors performed by bioinformatics analysis of IP-MS Menin partners list (Normal Prostate Cell for PNT1A, Androgen Independent Prostate Cancer Cell). **B** PNT1A, LNCaP, and PC3 cell lysates were used to immunoprecipitate (IP) Menin using anti-Menin or anti-immunoglobulin (IgG) antibodies. Total cell lysate (TCL) were extracted and blotted with anti-Menin, anti-SNW1, or anti-ERH antibodies.

We performed an IP-MS experiment in normal and cancer cells to identify Menin co-reguators. Over the elevated number of Menin partners found for PNT1A (84), LNCaP (216), and PC-3 (113) (Table S11), we focused our attention on partners involved in transcription regulation (Fig. 7, Table S12). In PNT1A (N) model, we found that the Menin tumor suppressor role is due to its interaction with the transcription factor SNW Domain Containing 1 (SNW1), which was previously reported to positively regulate the TGFb receptor signaling pathway, a key negative growth regulator in the normal prostate [32, 33]. On the other hand, in the cancer model (LNCaP and PC-3), Menin was found to interact with the enhancer of the rudimentary homolog (ERH) transcription factor (Table S12), recently reported to induce cell migration and invasion in urothelial carcinoma through c-myc activation [34]. Supporting this hypothesis, Wu et al. recently demonstrated that Menin enhances c-Myc-mediated transcription to promote cancer progression [35]. These results suggest that Menin and ERH co-activate c-myc to promote CRPC progression and PC treatment resistance. To validate the IP-MS results (Fig. 7A), we conducted IP experiments in normal and tumoral conditions. We found that Menin interacts with the transcription factor SNW1 in normal cells whereas it interacts with the transcription factor ERH in PC cells (LNCaP and PC-3) (Fig. 7B).

## Discussion

HSP27 belongs to the highly conserved family of small heat shock proteins and has been found as one of the most overexpressed proteins in CRPC. It has been shown to promote tumor growth despite low residual androgen levels *via* an interaction with a broad diversity of proteins [5]. An important function of HSP27 relies on its ability to stabilize many oncoproteins (i.e., chaperone activity), aiding in the progression of CRPC [6, 7]. In this study, we found that Menin is a new ubiquitin-proteasome-regulated HSP27 partner and highlights a new CRPC driving mechanism that can serve as a therapeutic target to restore PC treatment sensitivity.

Although Menin has been extensively characterized as a tumor suppressor in endocrine tumors [36], published data found that protein Menin is overexpressed in localized and mCRPC [21]. Protein overexpression was also previously observed in a small series of cases [21]. Here, we have strongly provided detailed information regarding Menin mRNA and protein expression in a large cohort of human databases (*N* = 2081) and TMA (*N* = 297). TMA analysis revealed that Menin expression increased in patients that received NHT treatment to become overexpressed in 90% of CRPC patients. Even if HSP27 does not impact the mRNA expression of Menin, analysis of transcriptional expression in clinical samples may provide clinically relevant information for this potential therapeutic target. Our present analysis including notably 1643 PC primary tumor and 90 PC metastatic samples showed that high Menin expression was associated with poor-prognosis factors such as metastatic stage, hormone resistance, and higher tumor grade, and was independently associated with worse BRFS and OS. These data support that Menin inhibition could represent a potential therapeutic opportunity for CRPC treatment.

Previously, small molecules targeting Menin-MLL interaction have been proposed as a therapeutic approach with promising results in leukemia [23, 37] and PC AR-positive models [21]. Although this approach is attractive for disrupting AR signaling pathway, it remains restricted to the AR pathway and Menin-MLL interaction. Furthermore, to affect gene expression, high concentrations of these compounds were required and the in vivo effect was relatively modest [38]. In the present study, we developed and patented the first Menin-specific inhibitor using ASO technology.

Here we demonstrate that Menin-ASO can inhibit AR-positive and negative CRPC growth in vitro and in vivo, to enhance chemotherapy sensitivity and can serve as an efficient tool to restore CRPC treatment sensitivity with a less toxic effect. Recently, we reported that Menin-ASO also delays tumor progression in triple-negative breast cancer (TNBC) [20]. Oligonucleotide-based therapeutics in oncology utilize their specific high binding affinity for key oncogenic mRNA targets that can be abnormally expressed or spliced. Several ASOs have been recently approved for treating metabolic or neuromuscular human diseases [39], demonstrating the therapeutic potential of ASO in human disease. Furthermore, recent bioengineering innovations such as nanomedicine have improved efficacy, safety, and delivery of antisense oligonucleotides [40]. These improvements will undeniably improve the anti-cancer effect of these therapeutic agents.

To better understand how the Menin tumor suppressor can switch from suppressive to oncogenic functions in CRPC, we profiled Menin chromatin occupancy using ChIP-seq at different stages of the disease using normal (PNT1A), ASPC (LNCaP), and AIPC (PC-3) models. Bioinformatics analysis using KEGG pathways enrichment, shows that Menin plays a tumor suppressor role in normal prostate model PNT1A probably through microRNAs regulation (e.g., MIR1-2; MIR16-1; let-7d) known for their tumor suppressor roles in PC [41–44]. This result was supported by GO-BP enrichment, showing that Menin’s regulation leads to cell cycle blockade. In the AIPC model, bioinformatics analysis using ChIP-seq combined with RNA-seq data showed that Menin activates genes (RTKs, GTPases, RAS, and G protein-coupled receptors) involved in the PI3K/AKT signaling pathway largely known to be associated with disease progression, resistance to castration and poor outcome in CRPC [45]. Our data also suggest that in AIPC condition, Menin activates the PI3K/AKT alternative oncogenic pathway and promotes treatment resistance through activation of the oncogene MET, which was reported as a key participant in resistance mechanisms for targeted therapies in multiple types of cancers [46]. The PI3K/AKT pathway was previously shown to induce drug resistance to docetaxel and cisplatin resistance in different cancers [47–49]. In addition, ChIP-seq KEGG analysis showed the involvement of Menin in platinum resistance exclusively in the AIPC model. All together, our data support the use of Menin inhibitor to restore sensitivity to different therapeutics such as castration, Cisplatin, tyrosine kinase inhibitors, and docetaxel can be considered in CRPC treatment.

**Fig. 8:**
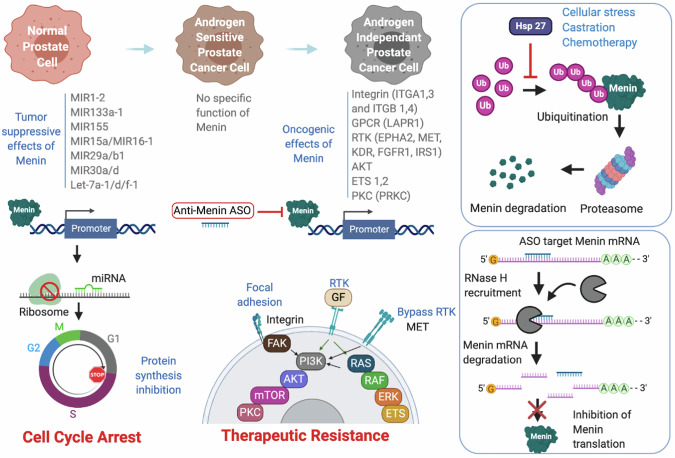
Menin switches from tumor suppressor to oncogenic role in prostate cancer. Schematic display illustrating how Menin switches from tumor suppressor role through miRNA regulation in normal prostate to oncogenic role in the AIPC AR-negative model, through activation of PI3K/AKT pathways and drive disease progression to CRPC and resistance to treatments (created using BioRender).

Overall, our study defines how HSP27 interacts with and stabilizes Menin to lead PC progression and treatment resistance through its oncogenic functions. Moreover, we show how Menin switches from a tumor suppressor regulating microRNAs in normal prostate to PI3K/AKT pathway activation in CRPC, leading to treatment resistance (Fig. 8). Our developed and patented Menin-specific inhibitor restored therapy sensitivity and could be used in combinational therapy to restore treatment sensitivity of CRPC.

## Methods

### Cell lines and culture conditions

DU-145, PC-3 were purchased from the American Type Culture Collection (Rockville, MD) and maintained in Dulbecco’s modified Eagle’s medium (Invitrogen, Cergy Pontoise, France) supplemented with 10% fetal bovine serum (FBS). LNCaP was kindly provided by the University of Virginia (Charlottesville, VA) and maintained in RPMI 1640 (Invitrogen, Cergy Pontoise, France) supplemented with 10% FBS. PNT1A cell line was kindly provided by Roswell Park Memorial Institute and maintained in RPMI 1640 supplemented with 10% FBS. Cells were maintained at 37 °C in a 5% CO_2_ humidified atmosphere. All cells were tested regularly for Mycoplasma contaminations.

### Western blot analysis and immunoprecipitation (IP)

WB analysis and IP experiments were performed as previously published [2] using Antibodies as indicated in the Supplementary methods.

### Immunofluorescence

Immunofluorescence analysis was performed as described before [6] on PC-3, LNCaP-HSP27, and LNCaP-Mock cells with 1:100 mouse anti-Menin monoclonal antibody (SC-390345, Santa Cruz Biotechnology, CA, USA) or 1:100 rabbit anti-HSP27 polyclonal antibody O/N at 4 °C. Secondary fluorescent goat anti-mouse Alexa Fluor 488 antibody or goat anti-mouse Alexa Fluor 546 (Thermo Fisher Scientific, Waltham, MA, USA) was added for 1 h at RT. Images were captured using a Zeiss 510 META fluorescence confocal microscope plan 40X/1.4 (Zeiss, Le Pecq, France). Analysis of focal colocalization was performed with Image Proplus 6 software (MediaCybernetics, Wokingham, UK) with an assignment of yellow for colocalized foci and green or red as non-colocalization.

### Design and synthesis of antisense oligonucleotides

To design the antisense oligonucleotides (ASOs) targeting the entire Menin mRNA, an R-based software was developed in our laboratory by Pascal Finetti (PDA16130, 2017) as previously described [20]. The program’s output gives information about the ASO list sequences with their GC level and genes list with significant similarity. Final selection was made manually, excluding ASOs showing similarity to other genes. HSP27-ASOs sequences were manufactured by ISIS Pharmaceuticals (Carlsbad, CA, USA) and supplied by OncoGenex Technologies (Vancouver, BC, Canada). Menin ASOs were manufactured by Pr. Philippe Barthélémy. The scramble control sequence was 5′-CGTGTAGGTACGGCAGATC-3′ and designated control-ASO.

### ASO transfection and treatment of cells with cycloheximide, MG132, and Cisplatin

ASO transfections were performed as previously described [20]. To study effects on Menin proteasome degradation, cycloheximide (10 µg/ml) and MG132 (10 µmol/l) were added in a replaced medium for 4, 24, and 48 h at the end of the second transfection with ASOs. Regarding the Cisplatin treatment, cells were transfected with Menin- or control-ASO and subsequently treated with dose-dependent concentrations (0.5, 1, 2.5, 5, 10, 25, 30, and 40 μmol/L) of Cisplatin during 48 h.

### Cell viability analysis

Cell viability analysis was performed with 3-(4,5-dimethylthiazol-2-yl)-2,5-diphenyl tetrazolium bromide (MTT) as previously described [7]. PC-3 cells were seeded in 12-well plates at 30,000 cells/well and transfected after 48 h with 100 nmol/l of Menin- or control-ASO. Cells were then treated for 24 h with 50 nmol/l (half-maximal inhibitory concentration, IC_50_) of Docetaxel (Sanofi-Aventis, France). MTT was added to each well (1 mg/ml), and the plates were incubated for 2 h at 37 °C. Supernatants were then removed and formazan crystals dissolved in Dimethyl sulfoxide (DMSO). Absorbance (595 nm) was evaluated using a Sunrise microplate absorbance reader (Tecan, Männedorf, Switzerland). Each assay was performed in triplicate. Cell viability was expressed as the percentage of transfected cells absorbance compared to untreated cells.

### Flow cytometric analysis

Flow cytometry of propidium iodide-stained nuclei was performed as previously described [6]. One million PC-3 cells were plated into 10-cm dishes and treated the day after with 100 nmol/l of Menin- or control-ASO. DNA content was determined by flow cytometry using an LSRII SORP (Becton Dickinson, France). Cell death was then measured using FlowJo software (Tree Star, Inc., Ashland, OR, USA). Each assay was performed in triplicate.

### Assessment of in vivo tumor growth

PC-3 cells (3 × 10^6^) were inoculated subcutaneously with 0.1 ml of complete culture medium in the flank region of 4-week-old male non-obese diabetic (NOD) scid gamma (NSG) mice (*n* = 30) generated in our laboratory. Mean tumor volume was similar in all groups before therapy. When PC-3 tumors reached 50 mm^3^, mice (*n* = 10) were randomly selected for treatment with Menin- or control-ASO. Additional mice (*n* = 5) were added to each group to evaluate the Menin-ASO effect on Menin expression after 1 week of treatment. After randomization, 12.5 mg/kg Menin- or control-ASO were injected intraperitoneally 5 times per week for 5 weeks. Tumor volume defined once weekly was calculated by the formula length × width × depth × 0.5236. Data points were expressed as average tumor volume levels ± SEM. P. Rocchi who supervised the animal experiments, obtained permission for animal experiments by ethical committee following European rules (APAFIS#26169-2020062314545490 v4, Direction générale de la recherche et de l'innovation (DGRI), 75231 Paris Cedex 05). Mice were maintained in the animal facility (agreement #13.2700).

### Chromatin immunoprecipitation (ChIP), sequencing, and data processing

Cells were treated with formaldehyde (1%) 15 min at RT. Cross-linking was stopped by addition of 1.25 M glycine followed by two washes in PBS. ChIP was performed as described previously with minor modifications [50]. After IP using anti-Menin antibody. DNA was purified using DNA I-Pure kit (Diagenode, Seraing-Belgium). Sequencing and data processing were done as described in Supplementary methods.

### Immunoprecipitation coupled mass spectrometry analysis for Menin interactome

The procedure was previously described [20]. The IP experiments were done with triplicate and subjected to MS analysis with three technical replications. To validate IP-MS experiment results IP experiments were performed using an anti-Menin antibody as described before followed by WB using Rabbit monoclonal Anti-ERH antibody ([EPR10830(B)] ab166620, Abcam, Paris, France) and Rabbit polyclonal NCOA62/SNW1 antibody (ab70827, Paris, France).

### Statistical analysis

Gel band densities were measured with ImageJ software (NIH, Bethesda, Maryland, USA). Statistical analysis was performed using the GraphPad Prism program (GraphPad Software, San Diego, USA). All results were expressed as mean ± SEM. Significance was assessed by a two-tailed Student’s *t*-test and one-way analysis of variance. *P ≤ 0.05 was considered significant, with **P ≤ 0.01 and ***P ≤ 0.001.

## Supplementary information

Supplemental Material

Table S1

Table S6

Table S8

Table S11

## Data Availability

ChIP-seq data are deposited in the NCBI Gene Expression Omnibus (GEO; https://www.ncbi.nlm.nih.gov/geo/) under the accession number GSE132827.
