# Correction: Menin inhibition suppresses castration-resistant prostate cancer and enhances chemosensitivity

**DOI:** 10.1038/s41388-025-03614-7

**Published:** 2025-10-28

**Authors:** Chaïma Cherif, Dang Tan Nguyen, Clément Paris, Thi Khanh Le, Thibaud Sefiane, Nadine Carbuccia, Pascal Finetti, Max Chaffanet, Abdessamad El kaoutari, Julien Vernerey, Ladan Fazli, Martin Gleave, Mohamed Manai, Philippe Barthélémy, Daniel Birnbaum, François Bertucci, David Taïeb, Palma Rocchi

**Affiliations:** 1 Predictive Oncology Laboratory, Centre de Recherche en Cancérologie de Marseille, Inserm UMR 1068, CNRS UMR 7258, Institut Paoli-Calmettes, Aix-Marseille University, 27 Bd. Leï Roure, F-13009 Marseille, France; 2 Laboratory of Biochemistry and Molecular Biology, Science University of Tunis, 2092El Manar, Tunis, Tunisia; 3 The Vancouver Prostate Centre, University of British Columbia, Vancouver, BC Canada; 4 ARNA Laboratory, INSERM U1212, CNRS UMR 5320, University of Bordeaux, F-33076 Bordeaux, France; 5 Biophysics and Nuclear Medicine Department, La Timone University Hospital, European Center for Research in Medical Imaging, Aix-Marseille University, F-13005 Marseille, France

Correction to: *Oncogene* 10.1038/s41388-021-02039-2, published online 28 October 2021

In the original version, details about the animal ethics approval were not mentioned in the section ‘Assessment of in vivo tumor growth’. Accordingly the text passage now reads as follows:

‘P. Rocchi who supervised the animal experiments, obtained permission for animal experiments by ethical committee following European rules (APAFIS#26169-2020062314545490 v4, Direction générale de la recherche et de l’innovation (DGRI), 75231 Paris Cedex 05)’ instead of ‘Palma Rocchi possesses personal agreement (#A13-477) for the animal handling and experimentation for this study. Animal experimentation was carried out accordingly to ethics laws recommendations’.

Correction to: Competing Interests

In the original version, the ‘Competing Interest’ statement was incorrectly given as ‘The authors declare no competing interests’ but should have been ‘The University of British Columbia has submitted patent applications on Apatorsen, an antisense inhibitor of Hsp27, listing PR and MG as inventors. This IP has been licensed to OncoGenex Technologies, a Vancouver-based biotechnology company. PR and DT are cofounders of SilonTx ([Silon Therapeutics | Advancing Innovative Therapies | https://silontx.com/]), a biotech company started in April 2024 focusing on precision medicine and nucleic acid therapeutics’.

The original article has been corrected.

