# Supplementary material for: Menin inhibition suppresses castration-resistant prostate cancer and enhances chemosensitivity
Source: Oncogene. 2021 Oct 28;41(1):125–37. doi: 10.1038/s41388-021-02039-2 (PMC8724010; doi:10.1038/s41388-021-02039-2)
Supplement: Supplementary file 1 — Supplemental Material [file 41388_2021_2039_MOESM1_ESM.pdf]

SUPPLEMENTARY METHODS

Proteomics analysis

A large-scale proteomic approach was performed using a liquid chromatography-quadrupole time-of-flight mass spectrometry (LC-QTOF MS) approach as described previously (61) to analyze LNCaP-Mock and LNCaP-HSP27 proteins. Accumulated data was analyzed using Protein Lynx Global Server software (PLGS 2.3) with peptide and fragment mass accuracies of 15 ppm. This search engine was applied to the full Uniprot 15.0 database, human species. A search of SwissProt Homo Sapiens was also carried out using Mascot software search using PKL peak list files generated in PLGS. Ion score cutoff of 32 is specified as identical or highly homologous according to Mascot outputs. Peptide score ≥ 40 was considered a positive hits. Only proteins with positive peptide hits ≥ 2 were considered statistically significant.

Bioinformatics analysis of proteomics data

To evaluate HSP27 client protein's involvement in reported biological processes, pathways enrichment analysis on the identified proteins using KEGG Pathway database was performed. We used the R Bioconductor package "Pathview" to retrieve, integrate and visualize pathway enrichments.

Antibodies used for Western blot analysis and Immunoprecipitation (IP)

For western blot, we used 1:5000 rabbit anti-HSP27 polyclonal antibody (Enzo Life Science, Villeurbanne, France), 1:500 mouse anti-Menin monoclonal antibody (SC-390345, Santa Cruz Biotechnology, CA, USA), 1:2000 rabbit anti-Menin polyclonal antibody (Bethyl, Laboratories Inc, TX, USA) and 1:500 mouse anti-ubiquitin monoclonal antibody (Santa Cruz Biotechnology, Heidelberg, Germany). Loading levels were normalized using 1:2000 mouse anti-vinculin monoclonal antibodies (Sigma Chemical, St Louis, MO) or 1:2500 rabbit anti-

GAPH polyclonal antibody (Abcam, Paris, France). For IP, the supernatant was incubated with 8 μg/ml mouse anti-Menin monoclonal antibody (Santa Cruz Biotechnology, CA, USA) or mouse anti-IgG (as an internal control) O/N at 4°C. We used rabbit or mouse True Blot anti-rabbit IgG secondary antibody (Rockland, inc, Limerick, PA, USA) to reveal western blot.

Tissue microarray experiments

Menin protein expression was assessed in Vancouver Prostate Center using two different Tissue MicroArrays (TMA) built from 291 patients (2 cores from each patient) with a total of 582 valid cores. The first "Gleason" TMA includes 198 patients from untreated radical prostatectomies specimen arrayed according to their International Society of Urological Pathology (ISUP2014) Gleason groups including benign prostate hyperplasia (BPH; n=83), G1 (n=64), G2 (n=78), G3 (n=53), G4 (n=69), and G5 (n=49). The second "NHT" TMA includes 94 patients exposed to neoadjuvant hormone therapy (NHT) before surgery includes Naïve (n=76), NHT treatment (n=42) and transurethral resection (TURP) of CRPC (n=70). Immunohistochemistry (IHC) staining was conducted by Ventana Autostainer Model Discover XT (Ventana Medical System, Tuscan, Arizona). Enzyme-labeled biotin-streptavidin system and solvent-resistant 3,3′-Diaminobenzidine (DAB) Map kit was used with 1/50 mouse anti-Menin monoclonal antibody (Santa Cruz Biotechnology, CA, USA). Digital images were obtained using the Leica SCN400 scanning system (Concord Leica Microsystems, Ontario, Canada), and scoring was performed visually by an anatomopathologist (Ladan Fazli). Nuclear immunoreactivity of Menin was evaluated using a 3-point scale scoring system by assigning zero for negative, one for weak to moderate, and two for strong staining intensity in the majority of tumor cells.

Menin mRNA expression correlates with prostate cancer progression

We perfoExpression analysis of *MEN1* mRNA expression in a pooled series of 2,081 clinical tissue samples publicly available, including 272 “normal” samples (normal tissue and benign prostate hyperplasia: BPH), 1,643 PC primary tumor samples, and 90 PC metastatic samples

RNA isolation, Reverse Transcription and Quantitative Real-Time Polymerase Chain Reaction (RT-PCR)

Total RNAs were extracted from PC-3 treated with OGX-427 or control-OGX-427 using RNeasy® Mini quit (Qiagen, Courtaboeuf, France). mRNA (1 µg) was reverse transcribed to cDNA using SuperScript II Reverse Transcriptase and Random Primers (Invitrogen). To amplify *MEN1* sequence, specific primers were designed as forward: 5'-CCCTCTACCACAAGGGCATT-3', reverse: 5'-TCCCGGCAGTAGTTGTAGTC-3' and probe sequence Tamra 5'-FAM-ACAGTGGCCGTGTCCGCCCA-3'. Quantitative reverse transcription PCR analysis was done on the CFX96™ Real-Time PCR Detection System. Expression levels of the GAPDH (TaqMan® GAPDH Control Reagents, catalog number 402869; Applied Biosystems) gene were used as an internal control.

Sequencing and data processing

ChIP-seq libraries were generated using the MicroPlex Library Preparation Kit (Diagenode, Seraing-Belgium) following the manufacturer's instructions. Quantification and qualification of the library were performed using a Qubit® 2.0 Fluorometer (Life Technologies, Carlsbad, CA, USA) and a TapeStation System (Agilent Technologies, Les Ulis, France). ChIP-sequencing was performed with a NextSeq500 sequencer (Illumina, San Diego, CA, USA) using a 75-nt single-end protocol. Computational analyses are developed in the supplemental experimental procedure. Profiles of Menin ChIP-seq were obtained by processing normalized bigwig files through the deepTools suite (v2.2.4 computeMatrix, plotProfile). All sequencing data were visualized using Integrative Genomics Viewer (IGV v2.3.92). Statistical software R

(version 3.5.1, https://www.r-project.org/) was used for integration between ChIPseq (Differentially Bound Genes, DBGs) and RNA-seq (Differential Expression Genes, DEGs) comparative analyses. The biological significance of DBGs was explored by Gene Ontology (GO) term enrichment analysis including biological process (BP) and molecular function (MF) based on Bioconductor packages enrichR (https://cran.r-project.org/package=enrichR). KEGG pathway enrichment analysis of DBGs or Differentially Expressed and Bound Genes (DEBGs) was performed with enrichR. A value of $p<0.05$ was considered statistically significant.

Computational analyses

Reads with a Phred quality score less than 30 were filtered out. For Menin ChIP-seq, reads were mapped to Homo sapiens genome assembly (GRCh37/hg19) using default parameters of Bowtie2 (v2.3.4.1) (1). Duplicate tags were removed and mapped tags were processed for further analysis. High confidence binding sites were determined using MACS2 peak caller (2): broad mode (broad-cutoff=0.01 -p 0.01). Inputs were used as controls. To get rid of noisy signal issues, peaks located less than 2500 bp from each other were stitched together and peaks smaller than 500 bp wide were removed. To link ChIP-seq data with gene expression, only peaks located at genes TSS (TSS defined from -2kb to +5kb) were filtered in using BEDTools intersect (v2.17.0) with a minimum overlap of 1-base (3). For ChIP-seq quantitative analysis, mapped tags were counted within TSS-intersected peak coordinates (defined previously by MACS2) using featureCounts (4) and then normalized as "reads per million mapped reads" using their respective library size.

For IGV visualization, mapped tags were converted into BigWig using the deepTools suite (v2.2.4) (bamCoverage) (5) and normalized by their library size using (scaleFactor argument).

For RNA-seq, PC3 and LNCaP expression tables were obtained from the publicly available Gene Expression Omnibus (GEO) repository (accession number: GSE59009) and merged. The

supervised analysis was carried out using Welch's t-test and FDR adjustment using p. adjust function.

For ChIP-seq and RNA-seq crossing, only Menin target genes (i.e. genes bound by Menin on their TSS) were analyzed. The biological significance of Differentially Expressed and Menin-Bound Genes (DEBGs) was explored by GO term enrichment analysis including biological process (BP) and molecular function (MF), based on Bioconductor packages clusterProfiler (6) (https://bioconductor.org/packages/release/bioc/html/clusterProfiler.html). KEGG pathway enrichment analysis of DEBGs was performed with clusterProfiler as well. A value of $P<0.05$ was considered statistically significant.

Antibodies used for western blot to validate the pathways activated by Menin in CR models.

Mouse monoclonal Anti-Akt3 (Clinisciences, Clone EE-M14, 1/500), mouse monoclonal Anti-Ets-1 (Clinisciences, Clone C-4, 1/500), Anti-PKC mouse monoclonal IgG2a Antibody (Clinisciences, clone MC5, 1/500) and mouse monoclonal Anti-MET (Clinisciences, Clone D-4, 1/500).

Mass spectrometry analysis for Menin interactome

The immunoprecipitated samples were stacked on NuPAGE™ 4–12% Bis–tris acrylamide gels according to the manufacturer's instructions (Invitrogen, Life Technologies). Protein containing bands were stained with Thermo Scientific Imperial Blue, cut from the gel, and following reduction and iodoacetamide alkylation, digested with high sequencing grade trypsin (Promega, Madison, WI, USA). Peptides extracts were concentrated and analyzed by Liquid Chromatography coupled to tandem Mass Spectrometry (LC-MS/MS) using an LTQ-Velos-Orbitrap (Thermo Electron, Bremen, Germany) connected to a nanoLC Ultimate 3000 Rapid Separation Liquid chromatography system (Dionex, Sunnyvale, CA). 10% of whole sample (5

µl) were injected in triplicate on the system. After pre-concentration and washing of the sample on a Dionex Acclaim PepMap 100 column (C18, 2 cm × 100 µm i.d. 100 A pore size, 5 µm particle size), peptides were separated on a Dionex Acclaim PepMap RSLC column (C18, 15 cm × 75 µm i.d., 100 A, 2 µm particle size) at 300 nL/min flow rate and eluted by a two steps linear gradient (4-20% acetonitrile/H20; 0.1 % formic acid for 90 min and 20-45% acetonitrile/H20; 0.1 % formic acid for 30 min. The separation of the peptides was monitored by a UV detector (absorption at 214 nm). For peptides ionization in the nanospray source, spray voltage was set at 1.4 kV at 275°C. All samples were measured in a data-dependent acquisition mode. Each run was preceded by a blank MS run to monitor system background. The peptide masses were measured in a survey full scan (scan range 300-1700 m/z at 30 K FWHM resolution at m/z=400, target AGC value of 1.00×10^6 and maximum injection time of 200 ms). In parallel to the high-resolution full scan in the Orbitrap, the data-dependent CID scans of the 10 most intense precursor ions were fragmented and measured in the linear ion trap (normalized collision energy of 35 %, activation time of 10 ms, target AGC value of 3.00×10^4, maximum injection time 100 ms, isolation window 2 Da). Parent masses obtained in Orbitrap analyzer were automatically calibrated on the 445.120025 ion used as lock mass. The fragment ion masses were measured in the linear ion trap to have maximum sensitivity and the maximum amount of MS/MS data. Dynamic exclusion was enabled with a repeat count of 1 and an exclusion duration of 30 s. Raw files generated from mass spectrometry analysis were processed with Proteome Discoverer 1.4.1.14 (Thermo Fisher Scientific). This software was used to search data via in-house Mascot server (version 2.4.1; Matrix Science Inc., London, UK) against the Human subset (20.202 sequences) of the SwissProt database (2019_05). Database search was done using the following settings: a maximum of two trypsin miscleavage allowed, methionine oxidation, N-terminal protein acetylation as variable modifications, cysteine carbamido-methylation as a fixed modification. A peptide mass tolerance of 6 ppm and a

fragment mass tolerance of 0.8 Da were used for search analysis. Only peptides with high stringency Mascot score threshold (identity, FDR<0,1%) were selected and used for protein identification. The "precursor ions area detector" option of Proteome Discoverer was activated to obtain MS area information of the 3 more intense peptides of the identified protein.

SUPPLEMENTARY FIGURES LEGENDS

Figure S1: (A) Quantification of Menin expression levels in LNCaP-HSP27 compared to LNCaP-Mock. Bands were quantified using ImageJ software normalized to vinculin protein levels. (B) Quantification of Menin expression levels in PC models LNCaP, DU-145 and PC-3. Bands were quantified using ImageJ software normalized to GAPDH protein levels (C) Quantification of Menin and HSP27 interaction in LNCaP-HSP27 compared to LNCaP-Mock. Bands were quantified using ImageJ software normalized to HSP27 protein levels. (D) Quantification of Menin expression levels after OGX-427 treatment. Bands were quantified using ImageJ software normalized to vinculin protein levels. Data were normalized to Control-OGX (100%) and are shown as mean ±SEM, n=3. Two-tailed, unpaired Student's t-test, *$P \leq 0.05$, **$P \leq 0.01$, ***$P \leq 0.001$. (E) Estimation of Menin half-life by a cycloheximide treatment in PC-3 cells. Cells were treated with cycloheximide (CHX) (10 µg/ml) or CHX followed by MG-132 (10 µmol/l) for indicated periods. Cells were harvested for protein extraction and evaluated by western blot analysis using anti-Menin and anti-vinculin antibodies.

Figure S2: (A) Inhibition of Menin enhances cell apoptosis specifically in AIPC (PC-3) model. PNT1A and PC-3 cells were transfected with 100 nMol Menin- or control-ASO for 2 days after the second transfection. Data were normalized to untreated cells, and are shown as mean ± SEM, n=3. Two-tailed, unpaired Student's t-test *$P \leq 0.05$, **$P \leq 0.01$, ***$P \leq 0.001$. (B) Quantification of Menin protein levels after ASO-Menin treatment *in vivo*. Bands were quantified using ImageJ software normalized to vinculin protein levels. Data were normalized

to Control-OGX (100%) and are shown as mean ±SEM, n=3. Two-tailed, unpaired Student's t-test, **P*≤0.05, ***P*≤0.01, ****P*≤0.001.

Figure S3: (A) Average profile of ChIP peaks is a graph showing the read count frequency in the range from –1000bp to +5000bp. (B) Volcano plot representing differential genes expression RNA-Seq in LNCaP compared to PC-3 (GSE59009). The y-axis corresponds to the mean expression value of negative log10 (adjusted p-value), and the x-axis displays the log2 fold change value. The yellow dots represent the up-regulated expressed genes (p-adj <0.01, log2 fold change >2). (C) The median effective concentration (EC50) of cisplatin is at 27.9 µM. PC-3 cells were transfected with 100 nM Menin- or Control-ASO for 2 days and treated at 0, 1, 2.5, 5, 10, 25, 30 or 40 µM of cisplatin for 48 hours after the second transfection. Cell viability was determined using MTT test.

SUPPLEMENTARY TABLES

Table S1. Protein list detected in LNCaP-HSP27 and LNCaP-Mock cells (MS Excel file).

Table S2. List of KEGG pathways where HSP27 is implicated.

KEGG Identifier	Name
hsa04210	Apoptosis
hsa05215	Prostate cancer
hsa04110	Cell cycle
hsa00620	Pyruvate metabolism
hsa04010	MAPK
hsa04151	PI3K/AKT
hsa04014	Ras
hsa05202	Transcriptional misregulation in cancer
hsa04370	VEGF
Hsa04115	P53 signaling pathway

Table S3. List of Menin isoforms detected in LNCaP-HSP27 compare to LNCaP-Mock.

Menin isoforms	Number of peptides detected	
	LNCaP-HSP27	LNCaP-Mock
O00255	6	0
O00255-2	5	0
O00255-3	5	0
E7EN32	5	0
E7EPR4	4	0
E7ET29	4	0
E7ENS2	3	0
Q9GZQ5	2	0

Table S4. Prostate_PublicSets-mRNA

References	Source of data	Technological platform	N° of probe sets/genes	N° of samples	Normal prostate samples (N)	Primary PC samples (N)	Metastatic PC samples (N)
(7)	GEO database, GSE55945	Affymetrix, array U133 Plus 2.0	54K	21	0	13	0
(8)	GEO database, GSE35988	Agilent, array 4x44K G4112F(014850)	44K	122	0	59	35
(9)	Array-Express database, E-MTAB-6128	Affymetrix, Human Gene 2.0 ST Array	54K	141	40	101	0
(10)	Array-Express database, E-TABM-26	Affymetrix, array U133 A+B	2x22K	57	13	44	0
(11)	GEO database, GSE10645	Illumina, DASL Human Cancer + Custom Prostate Panel	500+500 (1K)	596	0	596	0
(12)	BROAD Institute, Cancer Program portal https://portals.broadinstitute.org/cgi-bin/cancer/datasets.cgi	Affymetrix custom array, Hu6800 + Hu35KsubA	7K+9K	23	9	10	4
(13)	GEO database, GSE6811	Non-commercial spotted DNA/cDNA, YN Human 36K array	37K	35	0	24	11
(14)	GEO database, GSE21034	Affymetrix, Human Exon 1.0 ST Array	43K	185	29	131	19
TCGA, PRAD, Cell 2015	TCGA portal, https://tcga-data.nci.nih.gov	Illumina, RNA sequencing V2	25K	551	52	498	1
(15)	GEO database, GSE6099	Non-commercial spotted DNA/cDNA, Chinnaiyan	20K	104	18	32	20

		Human 20K Hs6					
(16)	GEO database, GSE6956	Affymetrix, array U133A 2.0	22K	89	20	69	0
(17)	GEO database, GSE68555	Affymetrix, array U95A+Av2+B +C	38K	157	91	66	0
Total				2081	272	1643	90

Table S5. Correlations of MEN1 mRNA expression with clinicopathological variables.

**Characteristics**		**N**	**"MEN1-low" class**	**"MEN1-high" class**	**p-value**
**Patients' age, median (range)**		1373	62.67 (37.3-83)	62.87 (45.98-78)	0,654
**ISUP2014 grade**					**1,03E-02**
	1	294	233 (29%)	61 (22%)	
	2	149	119 (15%)	30 (11%)	
	3	133	91 (12%)	42 (15%)	
	4	175	127 (16%)	48 (17%)	
	5	320	221 (28%)	99 (35%)	
**Serum PSA level (ng/ml), median (range)**		544	37.59 (0-3247)	16.02 (0-623)	0,105
**AJCC pathological tumor size (pT)**					**1,60E-04**
	pT2	686	551 (51%)	135 (39%)	
	pT3	712	512 (48%)	200 (58%)	
	pT4	21	12 (1%)	9 (3%)	
**AJCC pathological lymph node**					0,0625
	pN0	926	704 (86%)	222 (82%)	
	pN1	160	110 (14%)	50 (18%)	
**Surgical resection margins**					1
	R0	490	366 (67%)	124 (67%)	
	R1-2	239	178 (33%)	61 (33%)	
**Follow-up, months (range)**		1298	34 (1-219)	27 (1-194)	**3,28E-02**
**BRFS event**		1298	383 (39%)	140 (44%)	0,148
**10-year BRFS**		1298	42% [38-46]	34% [27-42]	**9,68E-03**
**OS event**		1222	135 (15%)	59 (20%)	**3,53E-02**
**10-year OS**		1222	83% [80-87]	74% [67-81]	**9,75E-04**

Table S6. Univariate and multivariate prognostic analyses for Biochemical Recurrence-Free Survival (BRFS) and Overall survival (OS) (MS Excel file)

Table S7. List of selected ASOs covering Menin mRNA.

ASO Number	Position	Antisense 5'-3'	% GC
**6**	211-230	CACCAAGGAAAGGAGCACCA	55
**7**	231-250	AAATCGTCCACGAAGCCCAG	55
**8**	251-270	TGACGCGGTTGACAGCCAGA	60
**9**	271-290	CTCGGGAACGTTGGTAGGGA	60
**12**	331-350	CACGGGAAAGTAGGTGAGGC	60
**13**	351-370	GGATAGAGGGACAGGTCGAC	60
**16**	411-430	GGATAGAGGGACAGGTCGAC	60
**17**	431-450	TGGAGACACCCCCTTCTCGA	60
**18**	451-470	CTTCTTCACCAGCTCACGGC	60
**19**	471-490	TTCCATATGACATCGGAGAC	45
**21**	511-530	GATGTGGGCCCGATCCTTGA	60
**22**	531-550	ATGAAGCTGAAGAGGGACTG	50
**23**	551-570	TGTCCAATTTGGTGCCTGTG	50
**27**	631-650	ATCCTCAGACAGGGCGAGGT	60
**28**	651-670	CCAAACACTACCCAGGCATG	55
**34**	771-790	TATGATCCTTTCAGGTACAG	40
**35**	791-810	TCTTGCGGTCACAGCGCATG	60
**36**	811-830	CACCATGTTCGCCACCTCCA	50
**37**	831-850	TGATGGCACAATGGAAGGGT	50
**39**	871-890	CTGCAGCTGCAGAAGCTCCA	60
**40**	891-910	AGCAGCCAGAGCAGCTTCTG	60
**41**	911-930	CCAGATGTCCCAGGTCATAG	55
**43**	951-970	TCTAGATCTGCCAGGTTCCC	55
**46**	1011-1030	GCAATGCCCTTGTGGTAGAG	55
**48**	1051-1070	GGGGTAGATGTGTTCATCCC	55
**50**	1071-1090	CATTGCGGTTGCGACAGTGG	60
**63**	1351-1370	GATGCCGTCGTAGAATCGCA	55
**64**	1371-1390	CTGCCCTCCTCCCATTTGCA	60
**66**	1411-1430	AAGAAAGGTGGCCCAGCCCA	60
**67**	1431-1450	AAACGGCCTAGGGACTGCAC	60
**69**	1471-1490	TCGGCTCACTATGCGCACCT	60
**86**	1811-1830	CGCTCGAGTTGATCTTGGTG	55
**87**	1831-1850	CGTGAGTTGCAGCTTGATGG	55
**88**	1851-1870	ATCTGCACTTGCGACTGTGC	55

Table S8. List of targets Menin detected using ChIP-seq (MS Excel file)

Table S9. List of microRNAs regulated by Menin in normal prostate model.

MicroRNAs	Targets	Roles	References
**MIR-1-2**	FN1 (fibronectin), LASP1 (LIM and SH3 protein 1) and PTMA (prothymosin alpha)	Inhibit cell adhesion and migration	(18)
**MIR-133a/b**	EGFR (epidermal growth factor receptor)	Inhibit proliferation, migration and invasion	(19)
**MIR-155**	CTHRC1 (collagen triple helix repeat containing 1)	Inhibit cell proliferation, cell cycle and promoted cell apoptosis	(20)
**MIR-15a, MIR16-1**	CCND1 (cyclin D1)	Inhibit cell cycle and proliferation	(21)
**MIR-29-a**	KDM5B (Lysine Demethylase 5B)	Inhibit cell cycle and promoted apoptosis	(22)
**MIR-29-b**	CDH2 (cadherin 2, type 1, N - cadherin (neuronal))	Inhibit cell adhesion	(23)
**MIR-30-a**	SIX1(Sine oculis homeobox homolog1)	Inhibit cell proliferation, and invasion	(24)
**MIR-30-d**	Src (tyrosine kinase)<br>NT5E (Ecto-5′-nucleotidase)	Inhibit cycle migration and invasion	(25), (26)
**Let-7-a-1**	E2F2 (E2F transcription factor 2)<br>CCND2 (Cyclin D2)	Inhibit cell cycle progression	(27)
**Let-7-d**	AEG1 (Astrocyte elevated gene-1)	Inhibit proliferation and invasion and promotes apoptosis	(28)
**Let-7-f**	AKT2 (protein kinase B)	Inhibit proliferation and promotes apoptosis	(29)

Table S10. List of the Differentially Expressed and Menin-Bound Genes (DEBGs) in PC-3 cell line. The genes in red were selected for biological validation.

ECM-receptor interaction	Focal adhesion	ErbB signaling pathway	Ras signaling pathway	EGFR tyrosine kinase inhibitor resistance	PI3K/AKT	Pathways in cancer	Transcriptional misregulation in cancer	Proteoglycans in cancer	Axon guidance
COL4A1	COL4A1	AREG	FGF5	NRG2	COL4A1	COL4A1	RUNX1	ITGB1	ITGB1
COL4A2	COL4A2	NRG2	VEGFC	**AXL**	COL4A2	COL4A2	JMJD1C	**FGFR1**	**EPHA2**
LAMA5	LAMA5	PIK3CA	**EPHA2**	**KDR**	LAMA5	LAMA5	HMGA2	**KDR**	**EPHA5**
ITGA1	ITGA1	SOS1	**FGFR1**	**MET**	ITGA1	ITGA3	HOXA10	**MET**	**MET**
ITGA3	ITGA3	**AKT3**	**KDR**	SOS1	ITGA3	ITGB1	SIX1	SOS1	PIK3CA
ITGB1	ITGB1	**PRKCA**	**MET**	PIK3CA	ITGB1	FGF5	SIX4	CD44	CAMK2D
ITGB4	ITGB4	CAMK2D	SOS1	**AKT3**	ITGB4	VEGFC	HHEX	PIK3CA	CAMK2G
CD44	MYL12A	CAMK2G	PIK3CA	**PRKCA**	AREG	**MET**	FUT8	**AKT3**	**PRKCA**
	VCL		**AKT3**		FGF5	**FGFR1**	PPARG	**PRKCA**	ABLIM3
	ACTN1		**ETS1**		VEGFC	SOS1	ETV5	CAMK2D	NRP1
	VEGFC		ETS2		**EPHA2**	EML4	PLAU	CAMK2G	NTN4
	**KDR**		**PRKCA**		**FGFR1**	PIK3CA	**MET**	CAV1	PPP3CB
	**MET**		RALBP1		**KDR**	**AKT3**		WNT7A	PPP3R1
	SOS1				**MET**	**PRKCA**		FZD8	SEMA3A
	PIK3CA				**IRS1**	**ETS1**		HIF1A	SLIT2
	**AKT3**				LPAR1	WNT7A		PLAU	SRGAP1
	**PRKCA**				SOS1	FZD8			
	CAV1				PIK3CA	BIRC5			
					**AKT3**	CAMK2D			
					**PRKCA**	CAMK2G			
					PPP2R5E	LPAR1			
						HIF1A			
						MECOM			
						RUNX1			
						PPARG			
						RALBP1			

Table S11. List of Menin interactors detected using IP-MS (MS Excel file).

Table S12. List of transcription factors interacting with Menin.

PNT1A	LNCaP	DU-145	PC-3
BCL11B	CBFA2T2	BCL11B	BCL11B
SMARCC1	**ERH**	**ERH**	**ERH**
SMARCE1	XPNPEP3	SMARCC1	CBFA2T2
XPNPEP3	**RNH1**	SMARCE1	**PWP1**
**SNW1**	**TRIM28**	PTRF	
**ARHGAP5**		**PRKCDBP**	
PTRF		**SDPR**	

SUPPLEMENTAL REFERENCES

1. Langmead B, Salzberg SL. Fast gapped-read alignment with Bowtie 2. Nat Methods. 2012 Mar 4;9(4):357–9.

2. Zhang Y, Liu T, Meyer CA, Eeckhoute J, Johnson DS, Bernstein BE, et al. Model-based analysis of ChIP-Seq (MACS). Genome Biol. 2008;9(9):R137.

3. Quinlan AR, Hall IM. BEDTools: a flexible suite of utilities for comparing genomic features. Bioinforma Oxf Engl. 2010 Mar 15;26(6):841–2.

4. Liao Y, Smyth GK, Shi W. featureCounts: an efficient general purpose program for assigning sequence reads to genomic features. Bioinforma Oxf Engl. 2014 Apr 1;30(7):923–30.

5. Ramírez F, Ryan DP, Grüning B, Bhardwaj V, Kilpert F, Richter AS, et al. deepTools2: a next generation web server for deep-sequencing data analysis. Nucleic Acids Res. 2016 08;44(W1):W160-165.

6. Yu G, Wang L-G, Han Y, He Q-Y. clusterProfiler: an R package for comparing biological themes among gene clusters. Omics J Integr Biol. 2012 May;16(5):284–7.

7. Arredouani MS, Lu B, Bhasin M, Eljanne M, Yue W, Mosquera J-M, et al. Identification of the Transcription Factor Single-Minded Homologue 2 as a Potential Biomarker and Immunotherapy Target in Prostate Cancer. Clin Cancer Res [Internet]. 2009 Sep 8 [cited 2020 Apr 8]; Available from: https://clincancerres.aacrjournals.org/content/early/2009/09/04/1078-0432.CCR-09-0911

8. Grasso CS, Wu Y-M, Robinson DR, Cao X, Dhanasekaran SM, Khan AP, et al. The mutational landscape of lethal castration-resistant prostate cancer. Nature. 2012 Jul 12;487(7406):239–43.

9. Kamoun A, Cancel-Tassin G, Fromont G, Elarouci N, Armenoult L, Ayadi M, et al. Comprehensive molecular classification of localized prostate adenocarcinoma reveals a tumour subtype predictive of non-aggressive disease. Ann Oncol Off J Eur Soc Med Oncol. 2018 01;29(8):1814–21.

10. Liu P, Ramachandran S, Ali Seyed M, Scharer CD, Laycock N, Dalton WB, et al. Sex-determining region Y box 4 is a transforming oncogene in human prostate cancer cells. Cancer Res. 2006 Apr 15;66(8):4011–9.

11. Nakagawa T, Kollmeyer TM, Morlan BW, Anderson SK, Bergstralh EJ, Davis BJ, et al. A Tissue Biomarker Panel Predicting Systemic Progression after PSA Recurrence Post-Definitive Prostate Cancer Therapy. PLOS ONE. 2008 May 28;3(5):e2318.

12. Ramaswamy S, Tamayo P, Rifkin R, Mukherjee S, Yeang C-H, Angelo M, et al. Multiclass cancer diagnosis using tumor gene expression signatures. Proc Natl Acad Sci. 2001 Dec 18;98(26):15149–54.

13. Tamura K, Furihata M, Tsunoda T, Ashida S, Takata R, Obara W, et al. Molecular Features of Hormone-Refractory Prostate Cancer Cells by Genome-Wide Gene Expression Profiles. Cancer Res. 2007 Jun 1;67(11):5117–25.

14. Taylor BS, Schultz N, Hieronymus H, Gopalan A, Xiao Y, Carver BS, et al. Integrative genomic profiling of human prostate cancer. Cancer Cell. 2010 Jul 13;18(1):11–22.

15. Tomlins SA, Mehra R, Rhodes DR, Cao X, Wang L, Dhanasekaran SM, et al. Integrative molecular concept modeling of prostate cancer progression. Nat Genet. 2007 Jan;39(1):41–51.

16. Wallace TA, Prueitt RL, Yi M, Howe TM, Gillespie JW, Yfantis HG, et al. Tumor immunobiological differences in prostate cancer between African-American and European-American men. Cancer Res. 2008 Feb 1;68(3):927–36.

17. Yu YP, Landsittel D, Jing L, Nelson J, Ren B, Liu L, et al. Gene expression alterations in prostate cancer predicting tumor aggression and preceding development of malignancy. J Clin Oncol Off J Am Soc Clin Oncol. 2004 Jul 15;22(14):2790–9.

18. Hudson RS, Yi M, Esposito D, Watkins SK, Hurwitz AA, Yfantis HG, et al. MicroRNA-1 is a candidate tumor suppressor and prognostic marker in human prostate cancer. Nucleic Acids Res. 2012 Apr;40(8):3689–703.

19. Tao J, Wu D, Xu B, Qian W, Li P, Lu Q, et al. microRNA-133 inhibits cell proliferation, migration and invasion in prostate cancer cells by targeting the epidermal growth factor receptor. Oncol Rep. 2012 Jun;27(6):1967–75.

20. Liu J, Chen Z, Xiang J, Gu X. MicroRNA-155 acts as a tumor suppressor in colorectal cancer by targeting CTHRC1 in vitro. Oncol Lett. 2018 Apr;15(4):5561–8.

21. Bonci D, Coppola V, Musumeci M, Addario A, Giuffrida R, Memeo L, et al. The miR-15a-miR-16-1 cluster controls prostate cancer by targeting multiple oncogenic activities. Nat Med. 2008 Nov;14(11):1271–7.

22. Li J, Wan X, Qiang W, Li T, Huang W, Huang S, et al. MiR-29a suppresses prostate cell proliferation and induces apoptosis via KDM5B protein regulation. Int J Clin Exp Med. 2015;8(4):5329–39.

23. Ru P, Steele R, Newhall P, Phillips NJ, Toth K, Ray RB. miRNA-29b suppresses prostate cancer metastasis by regulating epithelial-mesenchymal transition signaling. Mol Cancer Ther. 2012 May;11(5):1166–73.

24. Zhu Q, Li H, Li Y, Jiang L. MicroRNA-30a functions as tumor suppressor and inhibits the proliferation and invasion of prostate cancer cells by down-regulation of SIX1. Hum Cell. 2017;30(4):290–9.

25. Kao C-J, Martiniez A, Shi X-B, Yang J, Evans CP, Dobi A, et al. miR-30 as a tumor suppressor connects EGF/Src signal to ERG and EMT. Oncogene. 2014 May 8;33(19):2495–503.

26. Song Y, Song C, Yang S. Tumor-Suppressive Function of miR-30d-5p in Prostate Cancer Cell Proliferation and Migration by Targeting NT5E. Cancer Biother Radiopharm. 2018 Jun 1;33(5):203–11.

27. Dong Q, Meng P, Wang T, Qin W, Qin W, Wang F, et al. MicroRNA let-7a inhibits proliferation of human prostate cancer cells in vitro and in vivo by targeting E2F2 and CCND2. PloS One. 2010 Apr 14;5(4):e10147.

28. Li H, Zhao J. let-7d suppresses proliferation and invasion and promotes apoptosis of meningioma by targeting AEG-1. OncoTargets Ther. 2017 Oct 6;10:4895–904.

29. Li D, Chen L, Zhao W, Hao J, An R. MicroRNA-let-7f-1 is induced by lycopene and inhibits cell proliferation and triggers apoptosis in prostate cancer. Mol Med Rep. 2016 Mar 1;13(3):2708–14.

Supplementary Figures

A.

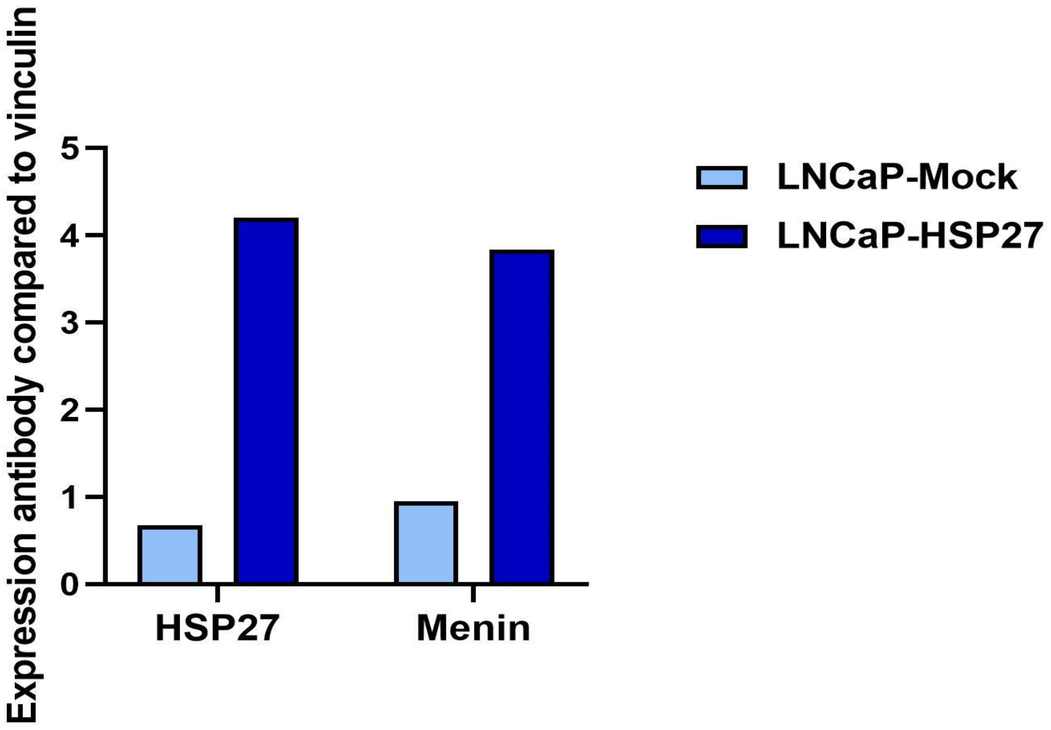

B.

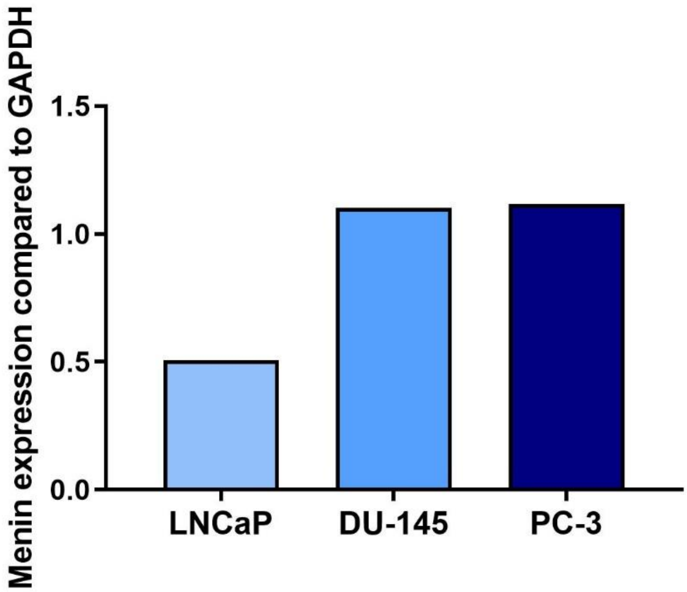

Figure S1

C.

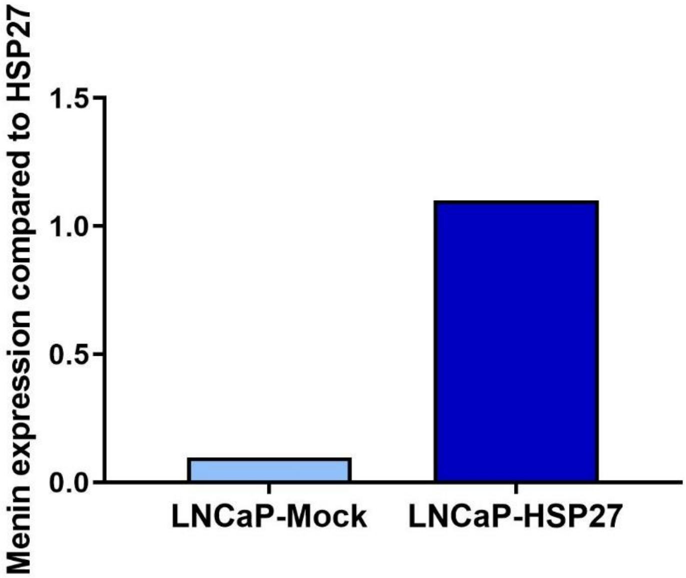

D.

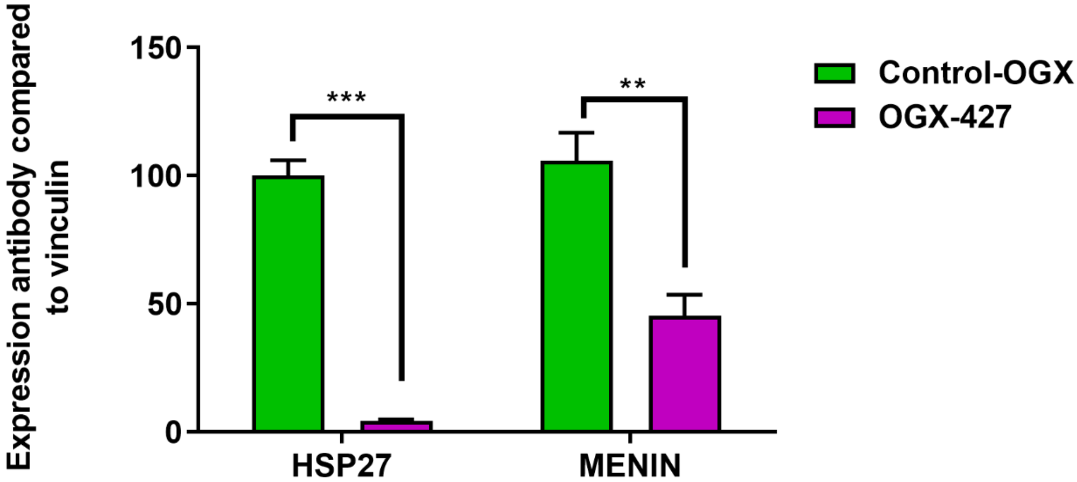

E.

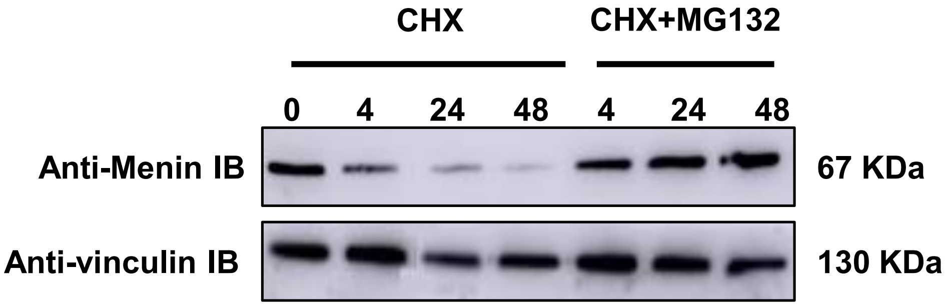

Figure S1

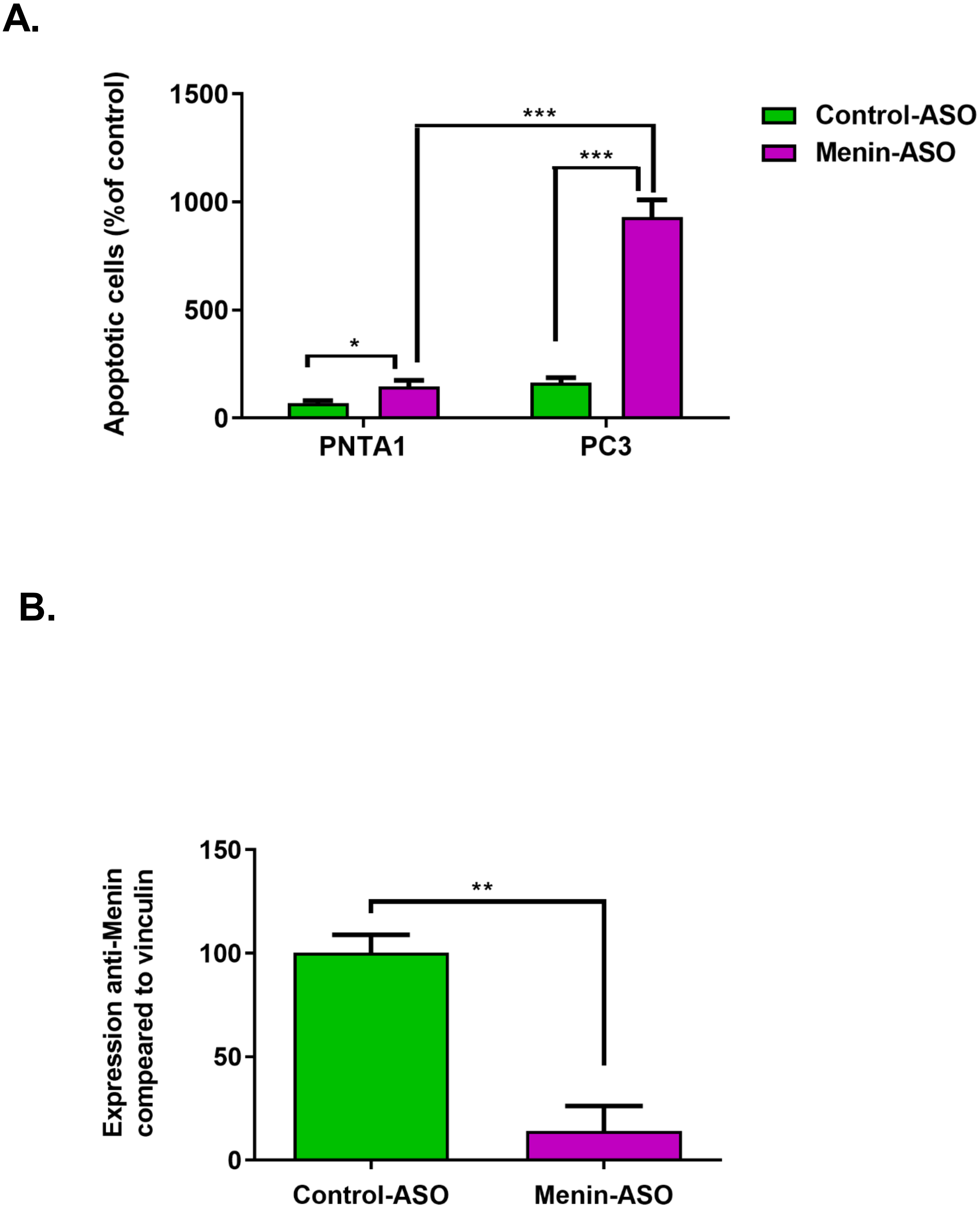
A.
Apoptotic cells (%of control)
1500
1000
500
0
***
***
*
Control-ASO
Menin-ASO
PNTA1
PC3
B.
Expression anti-Menin
compeared to vinculin
150
100
50
0
**
Control-ASO
Menin-ASO

Figure S2

A.

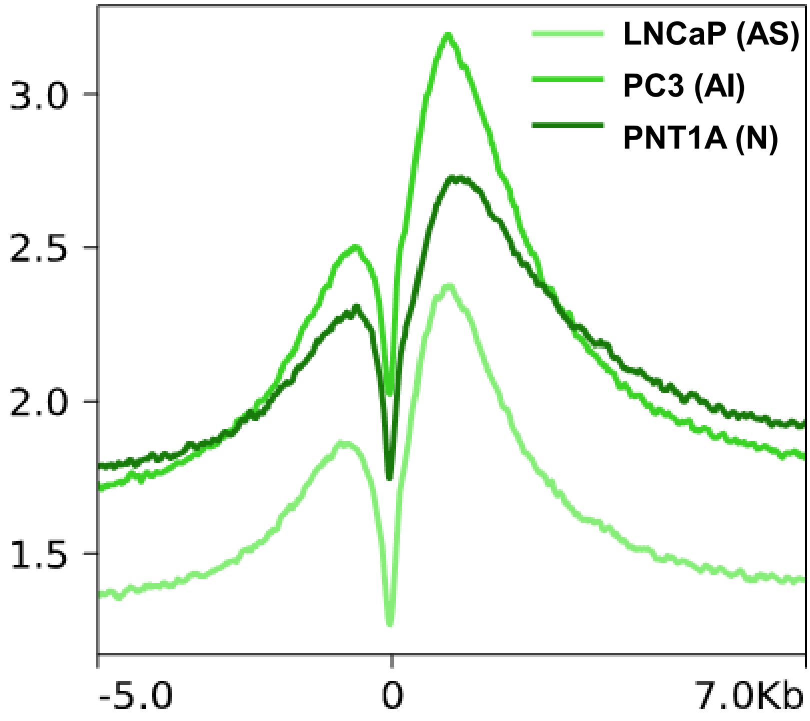

LNCaP (AS)
PC3 (AI)
PNT1A (N)
3.0
2.5
2.0
1.5
-5.0
0
7.0Kb

B.

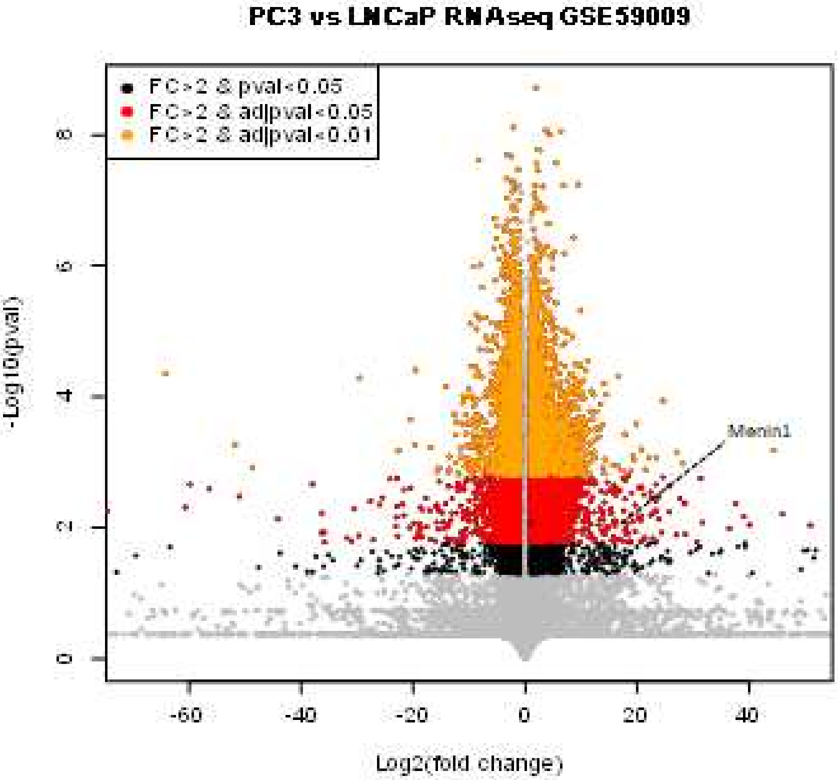

PC3 vs LNCaP RNAseq GSE59009
FC>2 & pval<0.05
FC>2 & adjpval<0.05
FC>2 & adjpval<0.01
Menin1
-Log10(pval)
Log2(fold change)
-60
-40
-20
0
20
40
0
2
4
6
8

Figure S3

C.

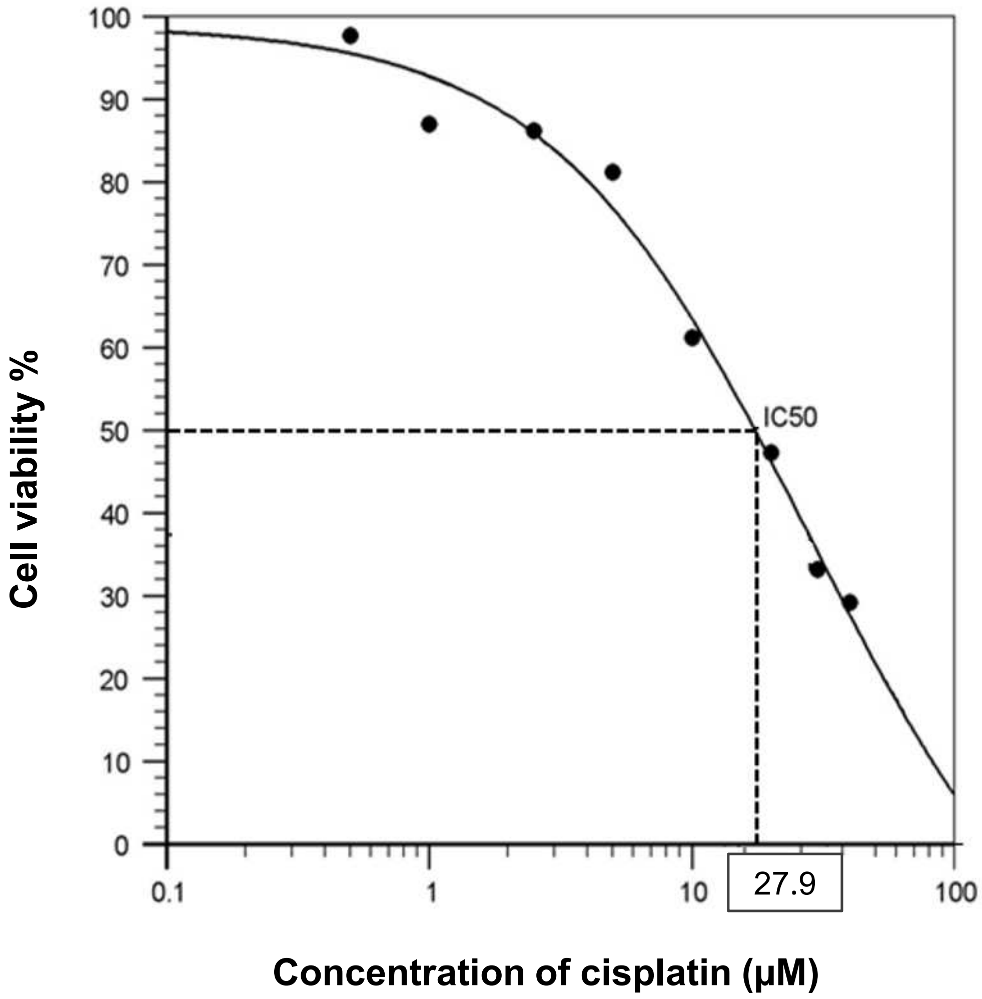

Cell viability %
100
90
80
70
60
50
40
30
20
10
0
IC50
0.1
1
10
27.9
100
Concentration of cisplatin (μM)

Figure S3
